# Molecular epidemiology and expression of capsular polysaccharides in *Staphylococcus aureus* clinical isolates in the United States

**DOI:** 10.1371/journal.pone.0208356

**Published:** 2019-01-14

**Authors:** Naglaa Mohamed, Yekaterina Timofeyeva, Dorota Jamrozy, Eduardo Rojas, Li Hao, Natalie C. Silmon de Monerri, Julio Hawkins, Guy Singh, Bing Cai, Paul Liberator, Shite Sebastian, Robert G. K. Donald, Ingrid L. Scully, C. Hal Jones, C. Buddy Creech, Isaac Thomsen, Julian Parkhill, Sharon J. Peacock, Kathrin U. Jansen, Matthew T. G. Holden, Annaliesa S. Anderson

**Affiliations:** 1 Pfizer Vaccine Research and Development, Pearl River, New York, United States of America; 2 The Wellcome Trust Sanger Institute, Cambridge, United Kingdom; 3 Vanderbilt Vaccine Research Program, Department of Pediatrics, Division of Pediatric Infectious Diseases, Vanderbilt University School of Medicine, Nashville, Tennessee, United States of America; 4 London School of Hygiene and Tropical Medicine, London, United Kingdom; 5 School of Medicine, University of St Andrews, St Andrews, Fife, United Kingdom; Universitatsklinikum Hamburg-Eppendorf, GERMANY

## Abstract

*Staphylococcus aureus* capsular polysaccharides (CP) are important virulence factors under evaluation as vaccine antigens. Clinical *S*. *aureus* isolates have the biosynthetic capability to express either CP5 or CP8 and an understanding of the relationship between CP genotype/phenotype and *S*. *aureus* epidemiology is valuable. Using whole genome sequencing, the clonal relatedness and CP genotype were evaluated for disease-associated *S*. *aureus* isolates selected from the Tigecycline Evaluation and Surveillance Trial (T.E.S.T) to represent different geographic regions in the United States (US) during 2004 and 2009–10. Thirteen prominent clonal complexes (CC) were identified, with CC5, 8, 30 and 45 representing >80% of disease isolates. CC5 and CC8 isolates were CP type 5 and, CC30 and CC45 isolates were CP type 8. Representative isolates from prevalent CC were susceptible to *in vitro* opsonophagocytic killing elicited by anti-CP antibodies, demonstrating that susceptibility to opsonic killing is not linked to the genetic lineage. However, as not all *S*. *aureus* isolates may express CP, isolates representing the diversity of disease isolates were assessed for CP production. While approximately 35% of isolates (primarily CC8) did not express CP *in vitro*, CP expression could be clearly demonstrated *in vivo* for 77% of a subset of these isolates (*n* = 20) despite the presence of mutations within the capsule operon. CP expression *in vivo* was also confirmed indirectly by measuring an increase in CP specific antibodies in mice infected with CP5 or CP8 isolates. Detection of antigen expression *in vivo* in relevant disease states is important to support the inclusion of these antigens in vaccines. Our findings confirm the validity of CP as vaccine targets and the potential of CP-based vaccines to contribute to *S*. *aureus* disease prevention.

## Introduction

*S*. *aureus* is a highly successful pathogen with the potential to cause a range of severe life threatening diseases (e.g. pneumonia, bacteremia, endocarditis and sepsis) and represents a public health burden [1–4]. Vulnerability to this pathogen is further highlighted by the prevalence of antibiotic resistance among *S*. *aureus* isolates, exemplified by the emergence of methicillin-resistant *S*. *aureus* (MRSA), which often necessitates the use of more complex treatment options. Initially, MRSA was primarily confined to healthcare settings, hence referred to as healthcare-associated MRSA (HA-MRSA) [2, 4, 5]; however, the epidemiology of MRSA changed at the turn of the century with the emergence and spread of MRSA isolates among individuals not previously exposed to healthcare settings. These community-associated MRSA (CA-MRSA) isolates are associated predominantly with skin and soft tissue infections (SSTIs), but have also been linked to clinical syndromes such as necrotizing pneumonia and severe sepsis [6]. In the US, clinical isolates of CA-MRSA have been dominated by a single genetic background, USA300, a pulsotype within sequence type 8 (ST8) and clonal complex 8 (CC8), as defined by multilocus sequence typing (MLST) [7, 8]. More recently, USA300 clones have emerged among healthcare-associated infections, superseding USA100 clone (associated with ST5) as a cause of bloodstream and nosocomial infections in some geographic locations [9, 10]. Furthermore, the contribution of methicillin-susceptible *S*. *aureus* (MSSA) to the burden of *S*. *aureus* disease has arguably been under-appreciated [11, 12]. Whereas MRSA infections are often associated with the spread of a limited number of clones, MSSA infections are caused by a wide range of genetically diverse isolates [13, 14]. The continuous evolution of *S*. *aureus* disease epidemiology, high mortality rates, and longer hospital stays associated with *S*. *aureus* invasive infections have necessitated the development of a vaccine to reduce morbidity and mortality attributed to this pathogen.

*S*. *aureus* capsular polysaccharides (CP) are virulence factors that can protect the pathogen from complement binding and subsequent phagocytic killing by neutrophils [15–17]. Although 13 putative capsular polysaccharides have been reported, only isolates that express capsular polysaccharide type 5 (CP5) or 8 (CP8) have been associated with disease [18]. Both polysaccharides consist of similar trisaccharide repeat units of *N*-acetyl mannosaminuronic acid (ManNAc), *N*-acetyl-l-fucosamine (l-FucNAc), and *N*-acetyl-d-fucosamine (d-FucNAc) [19] but differ in the linkages between the sugars and the sites of O-acetylation on the monosaccharide units [16]. Generally, *S*. *aureus* isolates contain the genetic machinery to express either CP5 or CP8, and the close association between CP type and clonal complex (CC) strongly suggests that these pathways are not subject to genetic switching to escape anti-capsular immunity [20, 21]. This is in contrast to other bacterial pathogens, which do utilize capsule switching, most likely to escape anti-capsular immunity (e.g. *Streptococcus pneumoniae* and *Neisseria meningitidis*) [22–24].

While the capsule can shield the pathogen from host immune defense mechanisms, it also presents a vulnerability to the pathogen since anti-capsular antibodies elicited by the host can opsonize the pathogen, facilitating uptake and killing by neutrophils [16]. Several highly successful vaccines to other bacterial pathogens target CP, including those against *Haemophilus influenzae* type b, *S*. *pneumoniae* and *N*. *meningitidis*, serogroups A, C, W, and Y [25–27]. Given these successes, *S*. *aureus* vaccine candidates based on CP have been tested clinically. To increase the immunogenicity of the CP and to induce T-cell memory, *S*. *aureus* CP5 and CP8 were conjugated to carrier proteins for use in preclinical and early clinical evaluations [28–30]. Preclinical studies with CP5 and CP8 antibodies or vaccination with CP conjugates showed some evidence of protection in animal models of *S*. *aureus* infection and that polyclonal antibodies to staphylococcal CP are opsonic for encapsulated *S*. *aureus* [28, 31–34]. Staphvax (by Nabi Biopharmaceutical), a first generation vaccine containing CP5 and CP8 coupled to detoxified recombinant *Pseudomonas aeruginosa* exotoxin A was the first to enter clinical efficacy phase 3 trials but failed to demonstrate efficacy in end-stage renal disease patients [35, 36]. The lack of efficacy of Staphvax raised concerns that *S*. *aureus* isolates may be deficient in CP expression, or that the patient population targeted was not competent in eliciting the appropriate protective immune responses. In addition, the vaccine didn’t consistently generate functional, bacterial killing responses in that patient population [35]. Furthermore, Scully et al. (2018) demonstrated that O-acetylation of the *S*. *aureus* capsular polysaccharide has to be maintained in the CP5 and 8 conjugates for them to induce bacterial killing antibodies [37]. Passive immunization has also been evaluated to prevent *S*. *aureus* infections. A Phase II trial of a polyclonal immune globulin (Ig) of anti-CP5 and anti-CP8 antibodies (Altastaph; by Nabi Biopharmaceutical) failed to show a significant difference in clinical outcomes among patients with complicated *S*. *aureus* bacteremia [38]. However, in another Phase II trial enrolled patients with documented *S*. *aureus* bacteremia who received Altastaph in addition to standard therapy or placebo, the vaccine induced CP antibodies were insufficient to significantly reduce *S*. *aureus* bacteremia [39]. Liu et al. (2017) attributed the inconsistency of protective efficacy of passive CP8 immunization to the release of solube CP8 by some *S*. *aureus* isolates, which can possibly interfere with antibody binding to the encapsulated bacterial surface [40–42]. Using lessons learned from previous clinical trials, the inclusion of multiple staphylococcal antigens would likely result in a more effective vaccine given the complexity of *S*. *aureus* and its myriad of virulence factors [43–45]. Thus, several next-generation multi-antigen vaccines containing CP conjugates are currently in development. The most advanced vaccine in clinical development is a 4 antigen *S*. *aureus* vaccine (SA4Ag) candidate that is being evaluated in a phase 2b/3 efficacy study [31, 46]. SA4Ag is composed of CP5 and CP8 individually conjugated to to the nontoxic mutant form of diphtheria toxin (cross-reactive material 197 [CRM197]), recombinant form of clumping factor A (rmClfA) and recombinant non-lipidated form of manganese transport protein C (MntC) [31, 47, 48].

Confirmation of antigen expression is important to support the inclusion of these antigens in a prophylactic vaccine. Due to the difficulty of studying bacterial gene expression *in vivo*, many studies have been performed under *in vitro* conditions to mimic the host environment, including nutrient limitation and low oxygen conditions [49, 50]. These studies have provided valuable information; however, the full complexity of the *in vivo* environment cannot be adequately mimicked. It has been reported that a significant proportion of clinical isolates do not express CP when grown *in vitro* [51–53]. This observation was first noted for isolates belonging to the USA300 lineage, and remained to be evaluated in other lineages [54, 55]. CP expression is highly regulated by complex genetic mechanisms and subject to *in vitro* and *in vivo* physiological signals, including those encountered in the host [56–63]. Recent data have emerged showing that a subset of *S*. *aureus* isolates that do not produce capsule *in vitro*, express the capsular polysaccharide when harvested from mice infected with these isolates [21, 31, 64]. To expand upon this observation, a prospective analysis of disease causing isolates collected in the US either in 2004 or 2009–10 as part of the T.E.S.T surveillance trial was performed to investigate CP genotype distribution and expression in the context of *S*. *aureus* epidemiology in the US.

## Materials and methods

### Ethics statement

For the experiments with blood from subjects vaccinated with Sa3Ag (3-antigen *S*. *aureus* vaccine, ClinicalTrials.gov NCT01018641), the protocol, informed consent, and all associated documents were approved by the Human Research Ethics Committees for each of the participating study sites: Site 001, Alfred Research and Ethics Unit; Site 002, Bellberry Human Research Ethics Committee; Site 003, Children's, Youth and Women's Health Services Human Research Ethics Committee; Site 004, Princess Margaret Hospital for Children Ethics Committee; Site 005, Royal Children's Hospital and Health Services District Ethics Committee. Written informed consent was obtained from all subjects prior to study entry. The clinical study was conducted in compliance with Good Clinical Practice (GCP) guidelines of the International Conference on Harmonisation of Technical Requirements for Registration of Pharmaceuticals for Human Use, the International Ethical Guidelines for Biomedical Research Involving Human Subjects, and the principles of the Declaration of Helsinki.

All *S*. *aureus* Isolates including those obtained from the CDC and the global collection of Tigecycline Evaluation and Surveillance Trial were not linked to their associated patient data.

All animal studies were conducted according to Pfizer local and approved global Institutional Animal Care and Use Committee (IACUC) guidelines. The animal protocol is PRL-2011-00102 and it was approved by the Pfizer, Inc., Pearl River IACUC. Pfizer, Inc., Pearl River utilizes the Guide for the Care and Use of Laboratory Animals (Guide, NRC, 2011) as the primary standard for all animals under its institutional animal care and use program and also complies with the United States Department of Agriculture’s Animal Welfare Act (AWA) and Animal Welfare Regulations “Blue Book” (USDA/APHIS, November 2013) for regulated species (this does not apply to this protocol). In all instances where Guide recommendations are different from AWA regulations for covered species, the mandated governing standard shall apply. Female, CD1 mice 8 to 12 weeks old (Charles River Laboratories) were used and were humanely euthanized 6 h post infection by slow fill inhalation of CO_2_ followed by cervical dislocation or thoracotomy as a confirmatory method. No adverse events occurred and animals did not experience more than momentary pain or distress.

### Isolate selection

We obtained 516 *S*. *aureus* isolates that were collected as part of the global Tigecycline Evaluation and Surveillance Trial (T.E.S.T), now ATLAS (Antimicrobial Testing Leadership And Surveillance). For the purpose of conducting a longitudinal assessment of *S*. *aureus* isolates associated with disease in the USA, we prospectively selected healthcare centers across 6 US census regions (East North Central, East South Central, Middle Atlantic, South Atlantic, West North Central, West South Central) that were participating between 2004 and 2010 (five sites). Additional seven sites active at the time of our study were included. A total of 516 *S*. *aureus* clinical isolates derived from 2004 (*n* = 117), 2009 (*n* = 162) and 2010 (*n* = 237) were collected and analyzed. In line with T.E.S.T requirements, the participating US hospital sites collected consecutive isolates from patients with a documented infection of nosocomial or community origin. Only one isolate per patient was permitted and all body sites were considered as acceptable clinical sources. *S*. *aureus* isolates were cultured from specimens of patients aged 0 to 95 in various clinical settings, with samples broadly defined as derived from either inpatient (medicine general, medicine ICU, pediatric general, pediatric ICU, surgery general, and surgery ICU) or outpatient wards (clinic, office, emergency room and nursing home-rehab) [65]. Culture identification and data management were coordinated by a single reference laboratory (Laboratories International for Microbiology Studies, International Health Management Associates [IHMA], Schaumburg, IL). For the purpose of our study, all US isolates were grouped into HA-SA (obtained from inpatient wards) and community-associated (CA-SA, obtained from outpatient wards) infections and categorized into four main types defined as wound, invasive (organism is cultured from synovial and pleural fluids, blood or bone), respiratory or other infections.

For the *in vivo* expression experiments, additional isolates from a contemporary US collection were also tested. These isolates were obtained from the Centers for Disease Control and Prevention (CDC) from a reference collection of 1,984 MRSA isolates collected in 2005–2006 by the Active Bacterial Core surveillance system [66]. The clinical and epidemiologic characteristics of the these isolates are described in Murphy et al. [67].

### Construction of a PFESA0119 Δ*cap5HIJK ermC* strain

All PCRs were performed with iProof High-Fidelity DNA polymerase (Bio-Rad, Hercules, CA), and all restriction endonucleases were purchased from New England Biolabs (Ipswich, MA). A Δ*cap5HIJK ermC* knockout cassette, consisting of two ~1.1 kb fragments (cap-1 and cap-2) flanking the erythromycin resistance gene, *ermC*, was constructed. The primer sets (with relevant restriction sites in bold font) and DNA templates used to amplify each of the fragments were: (i) cap-1: oLH291 (GCT GCT **CCC GGG** GAC AAT AGT TGG TAC AAG GCC)/oLH277 (GTA GCA TGT CTC ATT CAA TTG CTA TCC TCA TCG TCA TTT CC), *S*. *aureus* strain Newman genomic DNA; (ii) *ermC*: oLH278 (GGA AAT GAC GAT GAG GAT AGC AAT TGA ATG AGA CAT GCT AC)/oLH279 (CGA CGT CCT TTT TAT TAA TGA AAA CTG GTT TAA GCC GAC), pE194; (iii) cap-2: oLH280 (GTC GGC TTA AAC CAG TTT TCA TTA ATA AAA AGG ACG TCG)/oLH281 (GCT GCT **GTC GAC** TCT CGT GCA ATT CCT TAC TCG), *S*. *aureus* strain Newman genomic DNA. These three products were spliced together by amplification with oLH291 and oLH281, and the knockout cassette was cloned into pSPT181 [68] at the *Xma*I and *Sal*I sites. After electroporation and chromosomal integration into *S*. *aureus* strain RN4220, the final *Δcap5HIJK ermC* mutant strain was obtained after plasmid excision by secondary recombination. This mutation was transduced into *S*. *aureus* isolate PFESA0119 essentially as described elsewhere [69]. In the resulting erythromycin resistant transductants, deletion of *cap5HIJK* was confirmed by PCR. Mutants were shown to be of the expected strain lineage with a Qualicon RiboPrinter (DuPont, Wilmington, DE).

### Whole genome sequencing of *S*. *aureus* isolates

Isolated colonies of pure cultures of *S*. *aureus* were cultured in TSB in 2 mL 96-well blocks. Genomic DNA was isolated using the Qiagen QIAcube system according to manufacturer’s protocol. Tagged DNA libraries were created using a method adapted from a standard Illumina Indexing protocol [70]. Whole genome sequencing was performed on the Illumina HiSeq 2000 platform with 100 bp paired-end reads. The Illumina sequence data were submitted to the European Nucleotide Archive (ENA) under accession numbers ERS234133- ERS432212 (S1 Table).

#### Whole genome sequence data analysis

Annotated assemblies were produced as previously described [71]. Briefly, *de novo* assembly of whole genome sequences was performed using Velvet v1.2 [72] with Velvet Optimiser v2.2.5 (http://www.vicbioinformatics.com/software.velvetoptimiser.shtml)). Contigs were scaffolded with SSPACE [73] and sequence gaps closed using GapFiller [74]. The draft assemblies have been submitted to the ENA with accession numbers (ERS234133- ERS432212) provided in S1 Table. The assembled contigs were annotated using Prokka v1.11 [75] and *S*. *aureus* specific database from RefSeq [76]. *In silico* PCR was used to determine the SCC*mec* type amongst *mecA* positive MRSA isolates [77, 78].

#### Isolate typing

*S*. *aureus* isolates were characterized by MLST [79] and *spa* typing (based on DNA sequence analysis of the *S*. *aureus*-specific staphylococcal protein A (*spa*) gene) [80]. For the MLST, the allelic profiles were extracted from the assembled genomes using an in-house developed pipeline, MLST check (https://github.com/sanger-pathogens/mlst_check). Novel allelic profiles and novel allele sequences were submitted to MSLT database curator (http://saureus.beta.mlst.net) and new STs were assigned. For the *spa* typing, the *spa* gene was extracted by an *in silico* PCR using previously described primers (http://www.ridom.de/doc/Ridom_spa_sequencing.pdf) and the *spa* types were assigned using the spaTyper application (http://spatyper.fortinbras.us). Presence of genes of interest (*mecA*, *pvl*, *cap* operon) was analyzed using the Short Read Sequence Typer 2 [81]. The capsular polysaccharide type was determined based on the presence of the type-specific *capHIJK* genes. *In silico* PCR was used to determine the SCC*mec* type amongst *mecA* positive MRSA isolates [77, 78].

### Opsonophagocytic assays (OPA)

OPA were performed as previously described [16]. OPA titers of sera from ten subjects vaccinated with Sa3Ag (pre-vaccination bleed and 29 days post-vaccination bleed) [82] were determined against CP5 and CP8 isolates representing the four most prevalent clonal complexes. OPA titers are defined as the reciprocal of the highest serum dilution that kills 50% of the bacteria in the test. Each sample was tested in replicate and geometric mean titers with 95% confidence interval were calculated using data from the ten subjects and from two independent experiments. Negative samples were arbitrarily assigned a titer of 50, which is half of the lowest serum dilution tested in the OPA. The specificity of the OPA was determined by preabsorbing immune serum with homologous capsular polysaccharides CP5 (140 μg/mL) or CP8 (320 μg/mL). After overnight incubation at 4°C, anti-CP antibody depleted sera were used in the OPA described above. Due to limited availability of serum volume, the PFESA2069 isolate was tested with depleted sera from 7 out of 10 vaccinated subjects.

### Detection of capsule production *in vitro* by Luminex-based assays

As not all 516 isolates were tested in this assay, the proportion that may not express capsule *in vitro* was estimated by calculating the % CP-negative isolates within each *spa*/CC group tested and then extrapolating to the % of the total population (*n* = 516) based on the distribution of each given CC. For example, 47 isolates of CC8 (37% of the total *S*. *aureus* population) were tested for *in vitro* capsule expression and since 41 CC8 isolates (87% of CC8) were CP-negative, the % CP-negative CC8 isolates was estimated to be 32% of the total population. The Luminex assays used to detect capsule expression *in vitro* are based on several steps including polysaccharide sample preparation, generation of monoclonal antibodies, coupling of CP5 and CP8 antigens to Luminex MagPlex microspheres, and analysis of samples by singleplex liquid array system, Luminex 200.

#### Polysaccharide sample preparation

*S*. *aureus* isolates were grown overnight on Columbia salt agar (CSA) plates and isolated colonies were then resuspended in Dulbecco’s PBS (DPBS) buffer without Ca^2+^/Mg^2+^ to one OD_600_. The cell suspension was then autoclaved at 121°C for 30 min as previously described [83]. Cells were pelleted by centrifugation (3,000 x *g*, 15 min) and supernatants were used in the Luminex assays.

#### Monoclonal antibodies (mAbs)

CP5 and CP8 specific mAbs were generated by immunizing mice (Swiss Webster, BALB/c, or SJL) with polysaccharides conjugated to the CRM197 carrier protein. These polysaccharides were produced according to the methods as described in Fattom et al. [84]. The CP were conjugated to cross-reacting CRM197 (cross-reacting material 197), a nontoxic recombinant mutant of diphtheria toxin, in a conjugation process using 1,1′-carbonyldiimidazole/1,1′-carbonyl-di-(1,2,4-triazole) (described in US patent US 8568735 B2). Splenocytes were fused with the non-secreting myeloma cell line X63Ag8.653 to generate hybridomas that were screened for the appropriate serotype specificity. The CP5-specific mAb CP5-5-1 is of the IgG1 subclass and the CP8-specific mAb CP8-119-11 is of the IgG2b subclass. Both mAbs were purified on Protein A column. The mAbs were conjugated with phycoerythrin (PE) and purified on Protein A column by Chromaprobe, Inc. (Maryland Hts., MO).

#### Coupling of CP5 and CP8 to Luminex MagPlex microspheres

CP5 and CP8 conjugated to poly-lysine (CP5-pLL and CP8-pLL) were passively coated individually onto unique Luminex MagPlex carboxylated microspheres. CP5-pLL (50 ng/mL final) or CP8-pLL (12.5 ng/mL final) were added to a 1 mL bead suspension (1.25 x 10^7^ microspheres) in DPBS and incubated for 2.5 h at room temperature with mixing. After unbound antigen was washed, the microspheres were blocked and stored in DPBS containing 1% BSA and 0.05% NaN_3_.

#### Analysis of samples by singleplex liquid array system, Luminex 200

CP5 and CP8 detection assays were performed individually using an antigen competitive assay format. CP5 or CP8 coated microspheres in DPBS containing 0.05% Tween-20 and 0.02% NaN_3_ were added to wells (2,500 beads/well) of the assay plate (Costar 3912). CP5 or CP8 reference standards serially diluted 2.5-fold in DPBS containing 0.05% Tween-20 and 0.02% NaN_3_ were added in triplicate to wells to create a 12-point titration standard curve. The concentration range of the reference standards was 0.06–1500 ng/mL and 0.01–250 ng/mL for CP5 and CP8 respectively. A volume of 50 μL of unknown samples was tested in duplicate. Finally, 50 μL of mAb CP5-5-1-PE (600 ng/mL) or mAb CP8-119-11-PE (105 ng/mL) in DPBS containing 0.05% Tween-20 and 0.02% NaN_3_ were added to each well of the assay plate. The plate was covered and incubated with shaking at 300 rpm at room temperature for 60 min. The plates were then washed three times with DPBS containing 0.05% Tween-20 and 0.02% NaN_3_. After the final wash, 100 μL of DPBS containing 0.05% Tween-20 and 0.02% NaN_3_ was added to each well and the plates were read at high reporter setting using a Bio-Plex reader (Bio-Plex 200 systems; Bio-Rad Laboratories, Hercules, CA). The quantity of CP5 or CP8 polysaccharide in test samples was derived from the CP5 or CP8 reference standard curves fitted using 4-Parameter Logistic (4PL) nonlinear regression analysis.

### Bioinformatics analysis of the *cap* operon and SNP calling

Single nucleotide polymorphisms (SNPs) in the *cap* operon were detected by mapping the paired-end reads against *cap* operons of selected reference isolates of *S*. *aureus*, using SMALT version 0.7.4. (https://www.sanger.ac.uk/resources/software/smalt/). Different reference sequences were used for CP5 and CP8 isolates. Furthermore, for each CP type two different reference genomes were used to verify identified polymorphisms and only the consensus SNPs were further analyzed (those found by mapping to both reference genomes of the same CP type). The CP5 isolates were mapped against the *cap* operon from the ST22 HO 5096 0412 isolate (GenBank accession HE681097) [85] and the SNPs were verified by mapping against the ST5 N315 isolate (GenBank accession BA000018). The CP8 isolates were mapped against the *cap* operon of the ST1 MSSA476 isolate (GenBank accession BX571857) and the SNPs were verified by mapping against the M013 isolate (ST59, accession number NC_016928). *In silico* PCR extraction of individual genes for all analyzed isolates was used to confirm the mapping results and analyses listed above.

To determine the distribution of all identified *cap* polymorphisms in the context of *S*. *aureus* phylogeny, evolutionary relationships between all isolates were reconstructed. Whole genome SNPs were detected by mapping the paired-end reads against the *S*. *aureus* N315 reference genome using SMALT. The generated whole genome sequence alignment was curated to exclude accessory regions (mobile genetic elements identified from the N315 reference genome). Based on the core genome SNP alignment an approximately-maximum-likelihood phylogenetic tree was constructed with FastTree software using generalized time reversible (GTR) model with GAMMA method of correction for among site rate variation. The phylogenetic tree was annotated with the distribution of identified SNPs and indels (S3 Fig).

### Detection of capsule production *in vivo*

#### Immunofluorescence microscopy assay (IFA) for the detection of surface polysaccharides

IFA was used to detect *in vivo* expression in *S*. *aureus* isolates. *S*. *aureus* isolation from infected animals and immunofluorescence staining were performed as previously described [64]. The accuracy of IFA in confirming capsule expression was first demonstrated using three different *S*. *aureus* strains; wild type Reynolds (CP5), its isogenic CP5-negative mutant, and the CP8-expressing isogenic Reynolds derivative in which *cap5H*-*cap5K* was replaced with *cap8H*-*cap8K* of the *cap8* gene cluster [86]. The strains were tested in a blinded fashion with two independent experiments per strain. Bacteremia (intraperitoneal challenge with 7×10^7^ to 2×10^8^ CFU *S*. *aureus* per animal) and wound infection (a 1-cm incision is made in the right thigh muscle of mice then closed with a suture, and 5 μL of an *S*. *aureus* suspension (5 × 10^7^ CFU) was introduced into the muscle incision under the suture) models were used. Female, CD1 mice 8 to 12 weeks old (Charles River Laboratories) were used in the *in vivo* experiments Duplicate samples were tested, one with CP5 specific antibody and the other with CP8 specific antibody. After specificity of the CP5 and CP8 antibodies had been confirmed with the *S*. *aureus* isogenic derivatives of strain Reynolds, strains from the T.E.S.T collection were tested in duplicate using the same IFA procedure.

#### Genetic comparison of challenge inocula with the bacteria recovered from infected animals

Mice (2 mice per bacterial strain) were challenged with one of three USA300 strains (PFESA0119, PFESA0029 and PFESASA0021) or Reynolds. Blood samples were collected from animals at two time points; T0 (pre-challenge) and 6 h after infection. Challenge stocks used to inoculate mice as well as blood samples were plated on Tryptic Soy Agar plates with 5% sheep blood. No colonies were recovered from the pre-challenge blood samples. Five colonies from the respective bacterial inocula and 10 colonies recovered from mice challenged with Reynolds strain (5 colonies per animal) and 20 colonies from mice challenged with each of the USA300 strains (10 colonies per strain per animal) were subjected to whole genome sequencing for comparative analysis of genotypic features and *cap* operon analysis.

#### Detection of capsule transcripts *in vivo* by qRT-PCR

RNA expression of two *cap* operon genes (*cap5D*, *cap5E*) and regulators (*mgrA*, *sarA*, *agrA*, RNA III and *ccpA*) was evaluated in animals infected with *S*. *aureus* isolates. Determination of 16S rRNA expression was included to normalize RNA expression levels of target genes. The isolates tested represented lineages that had a mixture of CP-expression positive and negative *in vitro* phenotypes; Reynolds, CC8-USA300 (PFESA0029, PFESA0119 and PFESA0021) and USA500 (PFESA0065) strains. Approximately, 4x10^8^ CFU of mid-log phase bacterial cultures grown in TSB were used to infect 10–12 week old CD1 mice intraperitoneally (IP). T0 represents the bacterial challenge sample. Bacteria were harvested from ~ 1.5–2 mL of blood at 1 and 4 hr post challenge (2 mice per time point per isolate) and mixed with 2 volumes of RNAProtect Bacteria Reagent (Qiagen, Valencia, CA); the samples were processed immediately for RNA extraction where samples were mixed thoroughly by vortexing and incubated at room temperature for 10–30 min. Mixtures were centrifuged at 5,000 x g for 10 min and supernatants removed. Pellets were suspended in 500 uL of RNA Protect and homogenized with an Omni tissue homogenizer (TH) (Omni International, GA) assembled with disposable Soft Tissue Omni Tip plastic homogenizing probes. Homogenates were centrifuged at 5,000 x g for 10 min and supernatants removed. Pellets were suspended in 1 mL of RLT buffer/β-mercaptoethanol buffer (QIAgen RNeasy kit), homogenized and centrifuged as above. Pellets were suspended in 110 μL of 2X TE buffer and transferred to 2 mL tubes harboring 50mg of glass beads (Sigma). A volume of 385 μL of RLT/β-mercaptoethanol buffer were used to rinse the 15 mL tubes and combined with the 110 uL 2x TE. Bacterial lysates were generated by beating the tubes at 1/30 s for 10 min in a Tissue Lyser (Qiagen) and cleared by centrifugation at 14,000 rpm for 2 min. A volume of 450 μL of lysate were mixed with 250 μL100% ethanol and applied to an RNeasy column (QIAgen). RNA was purified according to the QIAgen RNeasy Kit instructions with on-column DNase treatment to remove genomic DNA. RNA was eluted twice in 40 μL RNase-free water, quantified on a Nanodrop and stored -80°C. RNA integrity was evaluated by Bioanalyzer 2100 (Agilent, Santa Clara, CA).

For *in vivo* transcript profiling, 10 μL of RNA was reverse transcribed using SuperScript Vilo Master Mix (Invitrogen, Carlsbad, CA) and the resulting cDNA was diluted 5 fold with RNase-free water. Real-Time PCR reactions were assembled by adding 4 μL of diluted cDNA, 400 nM forward and reverse primers, 200 nM fluorescent-labeled probe (Applied Biosystems, Foster City, CA) and 5 μL of Taqman Fast Advanced Master Mix (Applied Biosystems) in a total volume of 10 μL. Reactions were performed using the 7900HT Fast Real-Time PCR System (Applied Biosystems) under the following conditions: 30 s at 95°C and 40 cycles of 3 s at 95°C and 30 s at 60°C. In some instances, transcripts were measured in one-step real-time PCR reactions assembled with 4 μL of RNA, 400 nM forward and Reverse primer, 200 nM Fluorescently labeled probe, 9.5 μL of EXPRESS One-Step qRT-PCR SuperMix (Applied Biosystems) in a total volume of 15 μL. The AB 7900HT cycling conditions consisted of 15 min at 55°C for cDNA synthesis, 2 min at 95°C and 40 cycles of 15 s at 95°C and then followed by 1 min at 60°C. For each transcript, the number of copies was determined from standard curves generated using 10^1^ to 10^7^ copies of plasmid harboring the corresponding gene. Normalized expression of transcripts was then expressed as copies per 10^7^ copies of 16S rRNA.

### Competitive Luminex immunoassay (cLIA)

cLIA was used to measure the level of serum antibody titers against CP5 and CP8 antigens in animals challenged with USA300 isolates (PFESA0029 [CDC3], PFESA0119 and PFESA0119 Δ*cap5HIJK ermC*). CD1 mice (7–9 weeks of age, 10 per group) were challenged with three intraperitoneal injections of ~ 2x10^6^ CFU/animal in 0.5 mL at 0, 6 and 14 weeks and bled before vaccination and two weeks after the final inoculation. CP5 antibody responses were measured using a CP5 specific cLIA assay. CP5 and CP8 antigens were coupled to a distinct fluorescent Luminex microsphere. Antigen-coated microspheres were incubated overnight at 4°C with appropriately diluted serum samples, controls, or reference standard serum. Antigen-specific functional mouse mAbs were then added to the microsphere/serum mixture and bound mAbs were detected with R-Phycoerythrin (PE) labeled rat anti-mouse IgG1 secondary antibody (Southern Biotech) using a BioPlex reader (BioRad) to measure fluorescence. In this competitive assay, the magnitude of the fluorescent PE signal is inversely proportional to the amount of antigen-specific antibody in the sample.

CP8 immune responses were also measured in animals challenged with CP8 *S*. *aureus* isolates including five CC12 isolates (PFESA1194, PFESA1305, PFESA1405, PFESA1502, PFESA2455) and two CC188 isolates (PFESA2058 and PFESA2089). CD1 mice (7–9 weeks of age, 10 per group) were administered with three intraperitoneal injections of ~ 2x10^6^ CFU/animal in 0.5 mL at 0, 4 and 12 weeks and bled at week 27. CP8 antibody responses were measured using a CP8 specific cLIA assay as described above for CP5 cLIA assay.

### Statistical analyses

The categorical variables in the molecular epidemiology analyses were compared using either the χ^2^ or likehood ratio test. A *P* value of <0.05 was considered significant. The cLIA serology data were described with geometric mean titer (GMT) and corresponding 95% confidence intervals (CIs). The CIs were constructed by back transformation of the confidence limits computed for the mean of the logarithmically transformed assay data based on Student t distribution. *P*-values based on 2-sample t-test or Wilcoxon test were presented to identify potential differences in immunoglobulin concentration.

## Results

### Patient demographics

The demographics of the patients with *S*. *aureus* infections in 2004 and 2009–10 are shown in Table 1. The differences in % males (*P* = 0.462) and median age (*P* = 0.717) were not significant among patients from the two time periods.

**Table 1:** Patient demographics and characteristics of *S*. *aureus* isolates.

	2004		2009–10^b^		*P*-Value^c^
Ward source	Inpatient (*n* = 80)	Outpatient (*n* = 37)	Inpatient (n = 294)	Outpatient (*n* = 95)	
***Patient*** ***characteristics***					
**Male, %**	59	59	56	53	0.4622
**Median age, yrs**	50	50	53	53	0.7168
***Infection*** ***Characteristics***					
**MRSA, *n* (%)**	56 (70)	30 (81.1)	126 (42.9)	48 (50.5)	<0.001
**Infection type, *n***					0.1137
**Wound**	25	18	65	39	
**Respiratory**	20	1	103	1	
**Invasive**^a^	21	2	66	14	
**Other**	14	16	60	41	
***CP genotypes***					0.2987
**CP5, %**	71	86	70	73	
**CP8, %**	29	14	30	27	

^a^ Invasive infections include blood, pleural and synovial body fluids and bone infections.

^b^ Ten isolates collected in 2009–10 had an unknown ward source.

^c^ *P* values compare variables between the two time periods.

### Clinical characteristics and sources of isolates

In total, 516 *S*. *aureus* isolates, collected through the T.E.S.T program from 12 hospitals in 6 US census regions in 2004 (*n* = 117) and 2009–10 (*n* = 399), were analyzed. A list of the 516 isolates is shown in S1 Table. No ward information was available for 10 isolates collected in 2009–10. The epidemiological characteristics of the isolates are described in Table 1. The proportion of MRSA infections among all *S*. *aureus* infections in inpatient and outpatient wards combined was significantly higher in 2004 (74%, 86/117) compared to 2009–10 (45%, 179/399) (*P* < 0.001). The majority of *S*. *aureus* isolates evaluated were obtained from healthcare-associated *S*. *aureus* (HA-SA) infections (72%, 374/516) and approximately 25% of these isolates were derived from invasive infections.

### Population structure of *S*. *aureus* isolates

#### Clonal complexes

**Fig 1:**
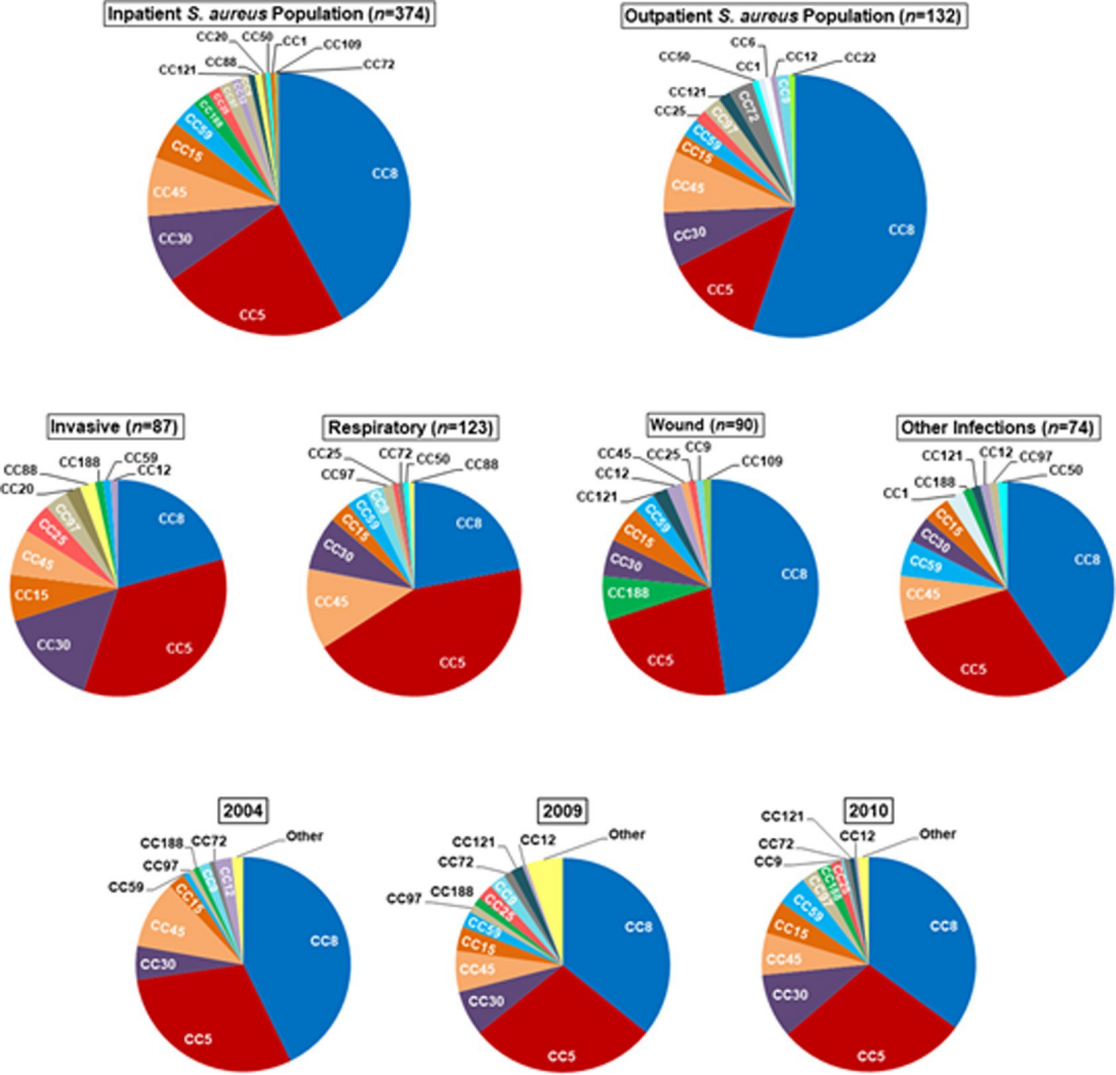
Clonal complex (CC) distribution among disease-causing *S*. *aureus* isolates in the US. (A) All isolates associated with *S*. *aureus* infections in healthcare and community settings. (B) *S*. *aureus* isolates associated with the main types of clinical infections in healthcare settings. (C). Temporal variation in the distribution of CC amongst *S*. *aureus* isolates.

The *S*. *aureus* population in the US, over the time period surveyed, included 20 clonal complexes (CC) comprised of 69 sequence types (ST, S2 Table). Overall, 13 CC represented 98% of disease causing isolates (Fig 1). Four of these CC represented >80% of isolates (Fig 1): CC8 (37%), CC5 (29%), CC30 (8%) and CC45 (7%). No clear distinction was observed between disease-causing CC in the healthcare setting compared to the community; four major lineages caused disease in both settings including CC5, 8, 30 and 45 (Fig 1A). There was a significant association between the isolate genotype (CC) and the type of clinical infection in inpatient wards (*P* = 0.036). For example, CC5 isolates were largely implicated in HA-SA invasive and respiratory infections, whereas CC8 isolates accounted for the majority of HA-SA wound infections (Fig 1B). The distribution of *S*. *aureus* isolates collected from inpatient wards at different census regions was reflective of the total *S*. *aureus* population with high prevalence of CC5 and CC8 isolates (S1 Fig).

The distribution of clonal complexes among disease isolates collected during 2004 (*n* = 117), 2009 (*n* = 162) and 2010 (*n* = 237) is illustrated in Fig 1C. In 2004, 13 CC were associated with disease, whereas 20 CC were associated with disease in 2009–10. For all time periods, CC8 and CC5 constituted the two most prevalent lineages followed by CC30 and CC45. While there was a trend in emergence of CC59 over time, the variation in the distribution of CC among 2004, 2009 and 2010 isolates was not statistically significant (*P* = 0.138).

#### *spa* type distribution

A total of 124 *spa* types were identified in 97% (*n* = 501 of 516) of isolates where a *spa* type was defined **(**S2 Table**)**. The distribution of prevalent *spa* types across the population in association with different infections reflected the CC distribution (S2 Fig). The most common *spa* types t008 (28%, 146/516) and t002 (20%, 105/516) were associated with the two dominant CC in the population, CC8 and CC5, respectively. CC30 was mostly associated with *spa* type t012, while CC45 had greater *spa* type diversity compared to other prominent CC where a single *spa* type dominated each lineage.

#### CC8-USA300 lineage

USA300 isolates belong to CC8, contain the SCC*mec* type IV element, and carry signature toxin genes such as Panton-Valentine Leukocidin (PVL) [87, 88]. The molecular epidemiology of CC8-USA300 isolates analyzed here was described previously [89]. Briefly, of the 191 CC8 isolates in the *S*. *aureus* population, 154 isolates (81%, 154/191) were typed as the USA300 clone. The majority of USA300 isolates (86%, 132/154) were genotyped as *spa* t008. The majority of these isolates (84%, 129/154) were MRSA. Most infections caused by USA300 isolates were associated with skin and wound infections (41%, 63/154). A relatively small number of USA300 isolates were obtained from invasive infections (7.8%, 12/154). The CC8 lineage also contained four representatives of the USA500 clone and six additional isolates closely related to USA500 but descending from a distinct node (USA500-like) [89]. All USA500 and USA500-like isolates belonged to *spa* type t064.

### Capsular polysaccharide genotype distribution

**Fig 2:**
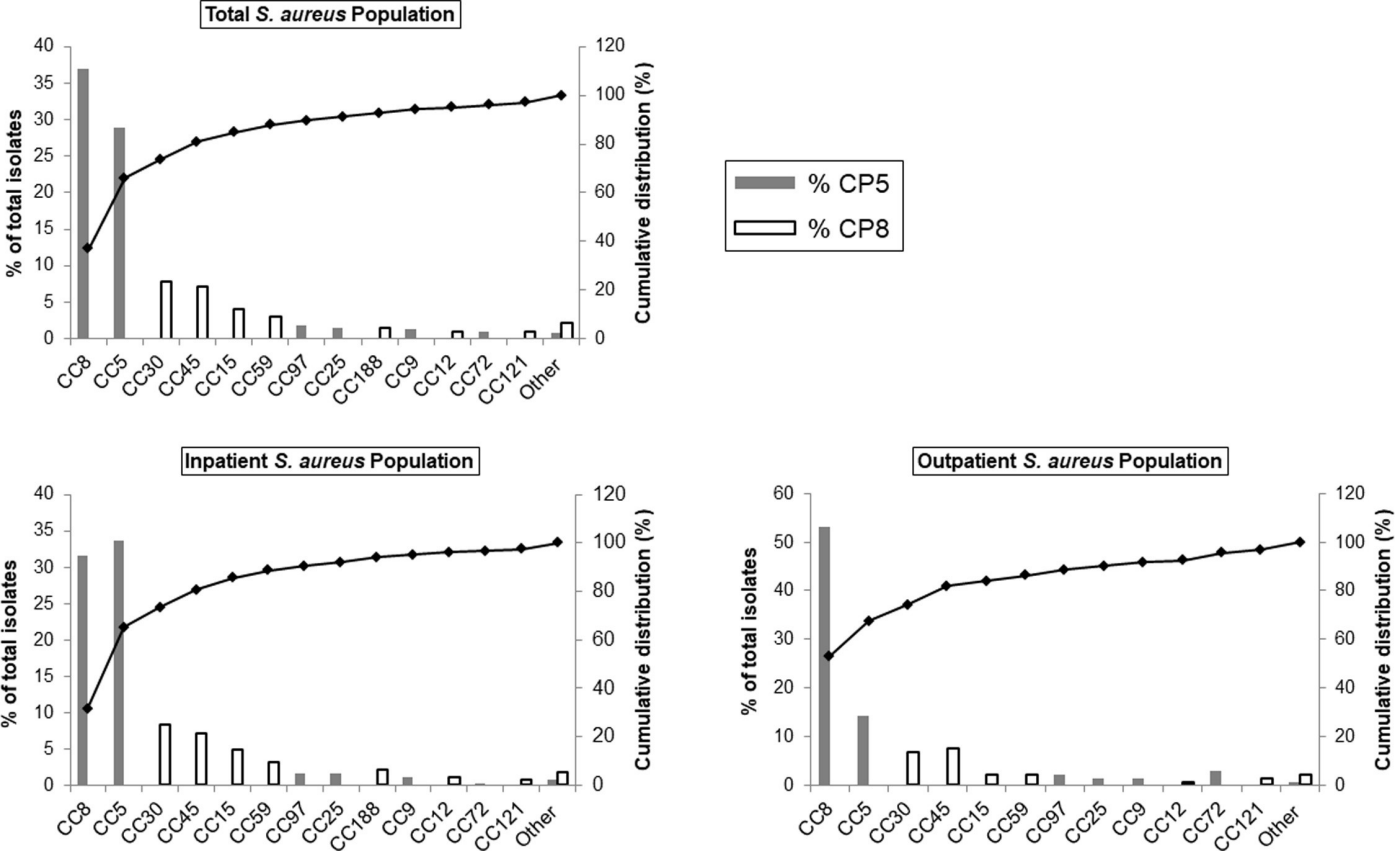
Distribution of clonal complexes (CC) and capsular polysaccharide (CP) genotypes by ward source.

Genotyping revealed that all study isolates possessed either *cap5* or *cap8* specific genes to direct the biosynthesis of CP5 or CP8, respectively (72% CP5 and 28% CP8). As previously reported [20], capsule genotypes were highly correlated with isolate lineage (Fig 2). Most MRSA isolates were CP5 (96%, 254/265), whereas MSSA isolates had a nearly equal distribution between CP5 and CP8 (S3 Fig). A CP5 genotype was significantly more prevalent among isolates collected in either time period from both inpatient and outpatient wards (*P* < 0.001) (Table 1 and Fig 2). The prevalence of CP5 isolates was higher among invasive isolates (67%, 70/105). The same trend was observed for other infection types where CP5 was the dominant genotype amongst clinical isolates. No association was observed between CP genotype distribution and either the type of infection (*P* = 0.724) or the ward source (*P* = 0.208).

### Opsonophagocytic killing of diverse clinical *S*. *aureus* isolates with immune sera

**Fig 3:**
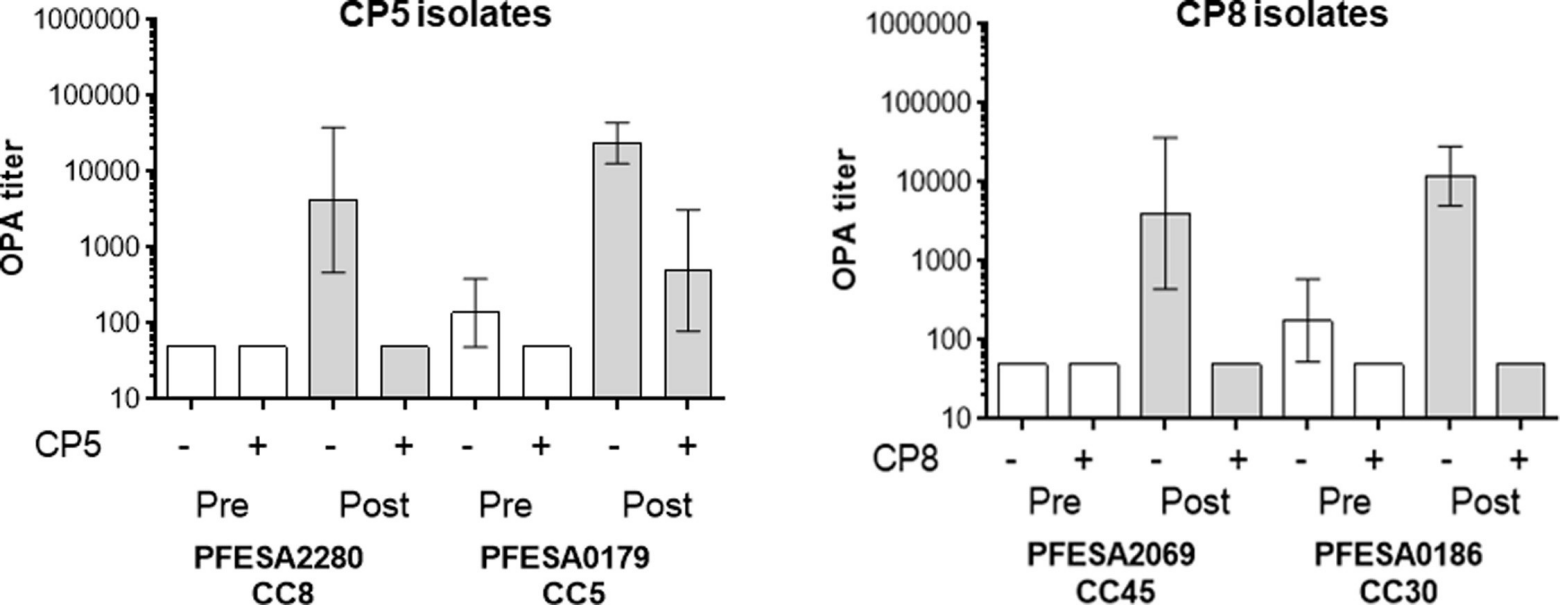
Killing of diverse *S*. *aureus* clinical isolates by human immune sera in CP5/CP8 OPA assays. Opsonophagocytic activity of clinical sera from ten vaccinated subjects (Pre: prevaccination bleed and Post: 29 days post vaccination bleed) were determined against CP5 and CP8 isolates representing the four most prevalent clonal complexes. Bars reflect geometric mean OPA titers from sera of ten immunized subjects tested in two separate experiments with 95% confidence intervals (error bars). CP specificity of the OPAs was demonstrated with anti-CP antibody depleted sera (CP5/8 +) generated as described in the Material and Methods section.

Clinical sera from human subjects immunized with an investigational vaccine (SA3Ag) containing CP5- and CP8-CRM197 conjugates [82] were evaluated for the ability to kill encapsulated *S*. *aureus* isolates in opsonophagocytic assays (OPA). OPA analyses of representative isolates from the four prominent CC (5, 8, 30 and 45), were conducted to evaluate the breadth of coverage of this investigational vaccine. An OPA titer was defined as the reciprocal of the sample serum dilution required to kill 50% of the bacteria in the test. Geometric mean OPA titers and 95% confidence intervals (CI) were calculated for samples (*n* = 10) tested in two independent experiments (Fig 3). OPA titers in sera from SA3Ag immunized subjects increased substantially compared to preimmune sera and for all isolates tested (Fig 3). Preabsorption of test sera with homologous capsule blocked the majority of vaccine elicited opsonic killing, illustrating the functional activity of antibody directed to the CP antigens.

### Detection of capsule expression *in vitro* by Luminex based assays

OPA demonstrated that antibodies elicited to the CP conjugate antigens contained in SA3Ag were able to kill diverse *S*. *aureus* clinical isolates. Since OPA are complex functional assays that are time consuming to develop for a large number of strains, CP5/8 detection assays were developed using a competitive antigen binding assay format to evaluate capsule expression *in vitro* for a larger panel of *S*. *aureus* clinical isolates. Initially, five randomly selected representatives from each of the 13 prominent CC among US disease causing isolates were tested. To account for the *spa* diversity within a given CC, additional isolates within each lineage were also evaluated (Table 2). All CP5 isolates tested were negative in the CP8 assay and *vice versa*. Among the representative CC 9, 15, 25, 30, 45, 59, 72 and 121 isolates tested, all expressed capsule *in vitro* (Table 2). However, CC5, 8, 12, 97 and 188 contained some isolates for which capsule production *in vitro* was below detectable levels (approximately 35% of the total isolates tested) (Table 2).

**Table 2:** *In vitro* and *in vivo* capsule expression by *S*. *aureus* isolates from major disease-causing clonal complexes (CC) and associated prevalent *spa* types.

CC (*n*)	CP genotype	*spa* types tested^a^ (n)	CP *in vitro* phenotype (*n*)	*cap* operon mutation type^b^ (*n*)	CP *in vivo* phenotype^c^ (*n*)
**CC8** (191)	**CP5**	t008^d^ (146)	POS (2)	No (1)	NT
				1 (1)	POS (1)
			NEG (21)	1, 2, 4 (2)	POS (1)
				1, 2, 3, 4 (19)	POS (13)
					NEG (5)
		t064^d^ (12)	POS (3)	No (1)	NT
				1 (2)	POS (2)
			NEG (14)	1, 2 (14)	POS (2)
					NEG (1)
		t024^d^ (9)	NEG (3)	1, 2, 4 (1)	NT
				1,2,3,4 (2)	POS (1)
		t121 (3)	NEG (2)	1, 2, 4 (1)	NT
				1,2,3,4 (1)	
		t681 (3)	NEG (1)	1,2,3,4 (1)	NT
		t334 (2)	POS (1)	No	NT
**CC5** (149)	**CP5**	t002 (105)	POS (4)	No	NT
		t045 (8)	POS (1)	No	NT
		t062 (8)	POS (1)	No	NT
			NEG (1)	5 (1)	
		t179 (3)	POS (2)	No	NT
		t306 (3)	POS (2)	No	NT
		t668 (1)	POS (1)	No	NT
**CC30** (40)	**CP8**	t012 (13)	POS (1)	No	NT
		t018 (4)	POS (1)		
		t021 (4)	POS (1)		
		t338 (4)	POS (2)		
		t2387 (1)	POS (1)		
**CC45** (37)	**CP8**	t553 (4)	POS (2)	No	NT
		t644 (4)	POS (2)		
		t004 (3)	POS (2)		
		t050 (2)	POS (1)		
		t671 (2)	POS (1)		
		t026 (1)	POS (1)		
		t1964 (1)	POS (1)		
		t2444 (1)	POS (1)		
**CC15** (21)	**CP8**	t084 (6)	POS (2)	No	NT
		t346 (3)	POS (2)		
		t605 (1)	POS (1)		
		t385 (1)	POS (1)		
		t094 (1)	POS (1)		
**CC59** (16)	**CP8**	t216 (13)	POS (2)	No	NT
		t437 (1)	POS (1)		
		t3736 (1)	POS (1)		
		t8419 (1)	POS (1)		
**CC97** (9)	**CP5**	t267 (4)	POS (1)	No	POS (1)
		t2802 (1)	POS (1)	No	NT
		t2297 (1)	POS (1)	No	NT
		t3380 (1)	POS (1)	No	NT
		t359 (1)	NEG (1)	Yes^e^	NEG (1)
**CC25** (8)	**CP5**	t078 (4)	POS (1)	No	NT
		t7084 (1)	POS (1)		
		t258(1)	POS (1)		
		t081 (1)	POS (1)		
		t1315 (1)	POS (1)		
**CC188** (8)	**CP8**	t189 (7)	POS (4)	No	NT
			NEG (1)	8 (1)	POS (1)
**CC9** (7)	**CP5**	t209 (5)	POS (4)	No	NT
		t193 (1)	POS (1)		
**CC12** (5)	**CP8**	t160 (2)	NEG (2)	6,7 (2)	POS (1)
					NEG (1)
		t156 (1)	POS (1)	No	POS (1)
		t5318 (1)	NEG (1)	No	POS (1)
		t771 (1)	NEG (1)	6,7 (1)	POS (1)
**CC121** (5)	**CP8**	NA (5)	POS (5)	No	NT
**CC72** (5)	**CP5**	t148 (2)	POS (2)	No	NT
		t1346 (1)	POS (1)		
		t3169 (1)	POS (1)		
		t1991 (1)	POS (1)		

^a^ Representative isolates spanning the *spa* diversity within each lineage were tested for CP expression.

^b^ The type of mutations in *cap5/8* operons are described in Table 3.

^c^ Results are only shown for isolates randomly selected and tested for *in vivo* capsule expression and NT refers to the isolates that were not tested for *in vivo* capsule expression.

^d^ Additional isolates associated with these *spa* genotypes from contemporary collections were tested for capsule expression.

^e^ The single CC97 isolate belonging to *spa* type t359 had a 1448 nucleotide deletion in *cap5D-E*.

### Genetic analysis of the *cap5/8* biosynthetic operon

To investigate the potential genetic basis for the lack of *in vitro* capsule expression in some isolates, whole genome sequencing data corresponding to the *cap* biosynthetic operon was analyzed for each of the 516 *S*. *aureus* isolates. Nucleotide polymorphisms were identified relative to the *S*. *aureus* reference isolates HO 5096 0412 (ST22) for CP5 isolates and MSSA476 (ST1) for CP8 isolates. Substantial single nucleotide polymorphism (SNP) diversity was detected within both CP5 and CP8 operons (S4 Fig). A small number of insertion/deletion (indel) mutations were also observed (S3 Fig). Most of the SNPs were detected in isolates that expressed capsule *in vitro*, suggesting that they do not negatively impact capsule production *in vitro*. For the SNP analysis of CC8 isolates, we focused on previously identified mutations that were linked to the CP-negative phenotype; whereas for the other CC, we screened the entire *cap* operon to correlate the occurrence of SNPs and the observed CP-negative phenotype. When compared with the respective reference isolates, a total of 8 different mutation types were identified among the isolates that lacked detectable capsule expression *in vitro* (Table 3). For CC8 isolates, four different mutations in the *cap5* operon were identified and *in vitro* negative CP phenotypes were associated with isolates that carried combinations of two or more of these mutations. These mutations have been previously described [55]. The *cap5* promoter had a transition mutation (T→C) positioned 73 nucleotides upstream of the ATG translation start codon of *cap5A* and was identified in 186 of 191 (97%) CC8 isolates. CC8 isolates with just this single mutation in the *cap5* promoter did express detectable CP5 *in vitro* as shown in Table 2. The majority of CC8 isolates (97%, 185/191) carried a single nucleotide insertion in *cap5D* (poly A heptamer occurring after nucleotide position 992) resulting in a translation frameshift and the introduction of a premature stop codon. This mutation was absent in the CC8 isolates where capsule was detected when cultured *in vitro*. The G→T transversion mutation at nucleotide position 223 of *cap5E* resulted in the non-synonymous substitution, Asp75Tyr, and was detected in all USA300 isolates (81% of the total CC8 isolates, 154/191), but not in other CC8 isolates. A T→C transition mutation at nucleotide position 478 in *cap5G* was detected in 89% (170/191) of CC8 isolates (including *spa* types t008, t024, t121 and t681) and resulted in the non-synonymous substitution, Phe160Leu. USA500 and USA500-like isolates (*spa* type t064) harbored the *cap5* promoter and the *cap5D* frameshift mutations, but the *cap5E and cap5G* mutations were not detected in these sub-lineages of *S*. *aureus* CC8 isolates.

**Table 3:** Summary of *cap* operon SNPs and indels detected in the study isolates typed as belonging to CC5, 8, 12 and 188.

Mutation type	CC	Gene	Nucleotide position in reference gene	Nucleotide in reference^a^	Nucleotide in study isolates (*n*/total)	aa in ref.^a^	aa in study isolates	Codon position	Percent of isolates
**1**	**CC8**	*cap5A*^*b*^	-73	T	C (186/191)	not applicable	not applicable	not applicable	97%
**2**	**CC8**	*cap5D*^*b*^	992	A	AA (185/191)	Lys	STOP^d^	338	97%
**3**	**CC8**	*cap5E*^*b*^	223	G	T (154/191)	Asp	Tyr	75	81%
**4**	**CC8**	*cap5G*^*b*^	478	T	C (170/191)	Phe	Leu	160	89%
**5**	**CC5**	*cap5E*^c^	340	C	T (1/149)	Gln	STOP^d^	114	1%
**6**	**CC12**	*cap8D*^c^	671	G	del (3/5)	Leu	STOP^d^	236	60%
**7**	**CC12**	*cap8E*^c^	683	G	T (3/5)	Ser	Ile	156	60%
**8**	**CC188**	*cap8O*^c^	690	G	T (1/8)	Gly	Val	251	13%

^a^ The reference isolates used for the genetic analysis of the *cap* loci were HO 5096 0412 for CP5 isolates and MSSA476 for CP8 isolates.

^b^ Previously described mutations in the *cap5* operon [55].

^c^ Novel mutations in *cap5/8* operons identified this study in association with *in vitro* CP-negative phenotypes.

^d^ The mutation results in truncated open reading frame.

The presence of capsule operon mutations was not restricted to the CC8 lineage of *S*. *aureus* isolates. In a single CC5-t062 isolate with a CP-negative *in vitro* phenotype, a premature stop codon was detected in *cap5E* (C→T resulting in a nonsense mutation). A different CC5 isolate of the same *spa* type that lacked this mutation was CP-positive *in vitro*. A similar genotype to phenotype correlation was observed for the CC188-t189 isolates. One isolate that did not express capsule *in vitro* had a unique SNP in *cap8O* resulting in a Gly251Val substitution. This SNP was not detected in four CC188-t189 isolates with a positive CP *in vitro* phenotype. Four of the five CC12 isolates did not express capsule *in vitro* and three of these shared a single nucleotide deletion in *cap8D* (position 671), resulting in a frameshift and premature stop codon. The same three isolates (two with *spa* type t160 and one with *spa* type t771) carried a G→T transversion mutation at nucleotide position 683 of *cap8E* resulting in a non-synonymous substitution, Ser156Ile. Interestingly, no specific mutations were detected in the fourth CC12 isolate (*spa* type t5318) with the CP-negative *in vitro* phenotype. A single CC97 isolate that did not express capsule *in vitro* harbored a 1448 nucleotide deletion involving the *cap5D* and *cap5E* genes.

### Detection of capsule expression *in vivo*

#### Detection of surface polysaccharides in the murine model by immunofluorescence assay (IFA)

**Fig 4:**
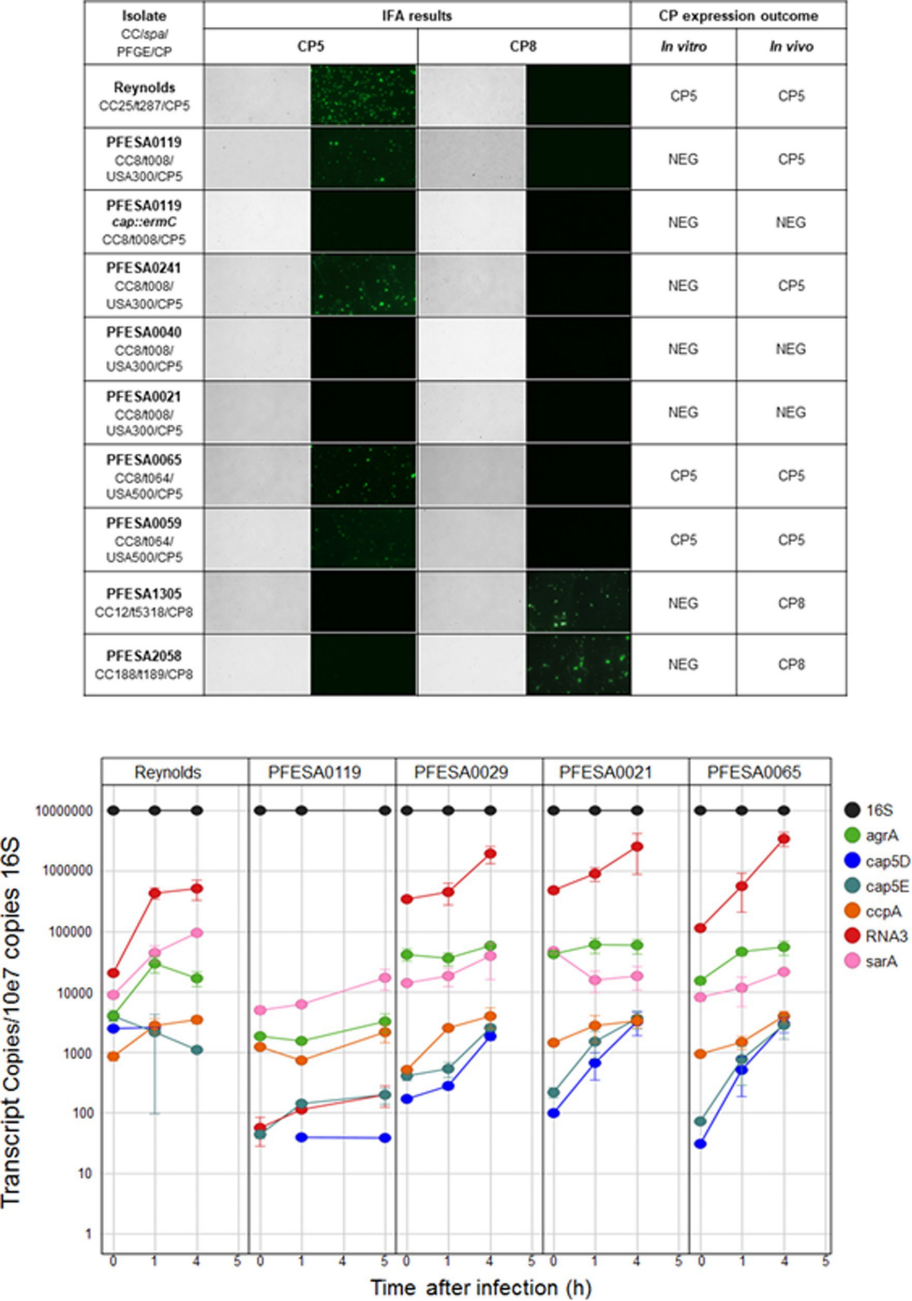
Detection of CP5 or CP8 polysaccharide expression in *S*. *aureus* isolates recovered from a murine bacteremia infection model. (A) Detection of surface polysaccharides in the murine IFA model. *S*. *aureus* was collected from the blood of infected mice 6h after infection, and stained with CP5 or CP8 antibodies. CP8 staining served as a specificity control for CP5-expressing isolates, and vice versa. As an additional control, the PFESA0119 Δ*capHIJK* isogenic deletion strain (PFESA0119 *cap*::*ermC*) was included. Isolate name, CC, spa type, pulsotype and capsule genotype are indicated next to each set of images. Both differential interference contrast (DIC) and IFA images are shown for each isolate. (B) *In vivo* transcript analyses of gene targets in four representative *S*. *aureus* CC8 isolates; three USA300 isolates (PFESA0119, PFESA0021 and PFESA0029) and one USA500 (PFESA0065) isolate. RNA levels corresponding to regulatory and biosynthetic CP genes (*agrA*, *cap5D*, *cap5E*, *ccpA*, *RNAIII* and *sarA*) were measured by RT-PCR in the bacterial challenge (T0) inoculum, as well as in mouse blood harvested at 1 and 4h after infection. Transcript copy numbers normalized to 16S rRNA are shown for triplicate samples at each time point.

To assess whether isolates with undetectable capsule expression *in vitro* may express capsule *in vivo*, IFA was used to test the majority of *S*. *aureus* isolates that were CP-negative *in vitro* [21, 64]. Blood was harvested from mice 6 h following infection with *S*. *aureus* strains, and evaluated by immunohistochemistry using anti-CP5 or anti-CP8 specific antibodies. The specificity of the anti-capsular polysaccharide antibodies used to detect capsule production by IFA was confirmed using three isogenic *S*. *aureus* Reynolds strains that expressed CP5, CP8, or no capsule (S5 Fig). The CP5 mAb only detected wild type *S*. *aureus* Reynolds, the prototype serotype 5 strain. Likewise the CP8 mAb only recognized the isogenic CP8 expressing Reynolds strain; neither antibody recognized the isogenic acapsular mutant strain. Of the five CC12 isolates tested, four (*spa* types t160, t5318 and t771) were CP-negative *in vitro* while one isolate (CC12-t156) was CP-positive. As discussed previously, the *in vitro* CP-negative CC12 isolates either had two SNPs in their capsule operon (*spa* types t160 and t771), or none (*spa* type t5318). CP8 expression was detected in blood following infection with three of the four *in vitro* CP-negative CC12 isolates (IFA data is shown for a representative isolate in Fig 4A). The CC12-t160 isolate that was CP-negative *in vivo* had mutations in *cap8D* and *cap8E* (mutation types 6 and 7, Table 3). The second CC12-t160 isolate with the same two mutations was positive for CP8 expression *in vivo*, despite having no detectable capsule expression *in vitro*. The single CC188-t189 isolate that was phenotypically CP-negative *in vitro* and harbored the non-synonymous Gly251Val substitution in *cap8O* (mutation type 8, Table 3) did express capsule *in vivo* (Fig 4A). The CC5-t062 isolate that was phenotypically CP-negative *in vitro* and contained the CP5 mutation (*cap5E*) resulting in a premature termination codon was not tested in the bacteremia model.

To more thoroughly investigate *in vivo* expression of CP5 in *S*. *aureus* CC8 isolates in light of contradictory conclusions in recent reports [21, 55, 64], additional CC8 *S*. *aureus* isolates from a contemporary US collection obtained from the CDC were included to supplement the CC8 isolates from the T.E.S.T collection. A total of 26 CC8 isolates were tested for capsule expression in mice, including 20 t008 isolates (17 of which are USA300 isolates), five t064 isolates (four of which are USA500 isolates), and one t024 USA300 isolate (Table 2). Three of the 26 CC8 *S*. *aureus* isolates in this subset expressed capsule *in vitro* (12%). As was seen with isolates from other lineages (e.g. CC12), no consistent association was found between the presence of mutations in the *cap* operon and the ability to express capsule *in vivo* (Table 2). Twenty of the 26 CC8 isolates tested expressed capsule *in vivo* (77%). Detection of capsule *in vivo* expression is shown for representative CC8-t008 (USA300) and CC8-t064 (USA500) isolates in Fig 4A.

To assess whether the observed *in vivo* expression was due to either mutations reverting *in vivo* or contamination by other *S*. *aureus* that are capsule producers, mice (two mice per bacterial strain tested) were challenged individually with three USA300 strains (PFESA0119, PFESA0029 and PFESASA0021) and Reynolds strain. For each of these strains, ST, *spa* type and *cap* operon sequences were identical in the challenge bacteria and the bacteria that were recovered from the animals after infection (S3 Table). There was no evidence of genetic changes or strain contamination.

Capsule polysaccharide expression *in vivo* was further explored by evaluating whether RNA transcripts from the *cap* operon could be detected in mouse blood following infection with a representative panel of USA300 (two *in vivo* CP-positive isolates, PFESA0119 and PFESA0029 [CDC3] and an *in vivo* CP-negative isolate, PFESA0021) and USA500 (*in vivo* CP-positive isolate PFESA0065) isolates. RNA transcripts corresponding to *cap5D and cap5E* were detected *in vivo* for each of the four isolates tested (Fig 4B).

**Detection of CP5 immune responses to USA300 isolates.** To further evaluate whether USA300 isolates express capsule *in vivo*, mice were infected with two *in viv*o CP-positive USA300 MRSA isolates PFESA0029 [CDC3] and PFESA0119 as well as the isogenic capsule knockout mutant (PFESA0119 Δ*cap5HIJK* ***ermC*)**. *S*. *aureus* (MSSA) strain Reynolds was used as a CP5 positive control. Sera were collected from mice before infection and 2-weeks after the final inoculation and were evaluated for the presence of CP specific antibodies. Antibody responses to CP5 were measured using a CP5-specific competitive luminex immunoassay (cLIA). Anti-CP5 antibodies were not detected in cohort mice prior to infection. However, a CP5 immune response was detected in mice infected with either of the USA300 isolates indirectly indicating CP5 capsule expression *in vivo*. No anti-CP5 antibodies were observed for the PFESA0119 capsule knockout mutant (Table 4).

**Table 4:** Demonstration of specificity of in vivo CP immune responses in mice challenged with *S*. *aureus* isolates. CP5 antibody titers in mice challenged with CP5 *S*. *aureus* isolates (MSSA strain Reynolds, two USA300 MRSA isolates PFESA0029 [CDC3] and PFESA0119, PFESA0119 *Δcap5HIJK ermC* capsule knock-out strains). CD1 mice (7–9 weeks of age, 10 per group) were administered three IP injections of ~ 2x10^6^ CFU/animal in 0.5 mL at 0, 6 and 14 weeks, and bled two weeks after the final inoculation. CP5 antibody responses were measured using a CP5 specific cLIA assay. CP5 antibody responses were detected in mice infected with strain Reynolds, and PFESA0029 and PFESA0119 isolates, while the capsule knockout mutant was not able to induce a CP5 antibody response. GMT fold change over the antibody titer of the capsule knockout mutant was calculated for all tested isolates.

		Challenge CP5 isolate			
CP5 cLIA titer	PFESA0119 Δ*cap5HIJK ermC*		PFESA0119	PFESA0029	Reynolds
Geometric mean titer (95% CI)		5 (5–5)	14 (5.9–33.3)	24 (8.8–66.8)	54 (10.7–274.8)
GMT fold change from baseline		1	2.8	4.8	10.8

CP8-specific cLIA was also used to evaluate immune responses to CP8 isolates that lacked capsule expression *in vitro*. Anti-CP8 antibodies were detected in mice infected with three CC12 isolates (PFESA1305, PFESA1405 and PFESA2455) and the CC188 isolate (PFESA2058) that harbored mutations in the *cap8* operon. No immune responses were detected in mice infected with the single CC12-t160 isolate (PFESA1502) that carried the same mutations in *cap8D* and *cap8E*, but was CP-negative *in vivo* (Table 5). These results are consistent with the IFA data and further support the observed *in vivo* capsule expression for CP8 isolates.

**Table 5:** Detection of CP5 and CP8 immune responses in mice challenged with *S*. *aureus* isolates. CP8 antibody titers in mice challenged with CP8 *S*. *aureus* isolates. A panel of seven different CP8 isolates representing different combinatins of *in vitro* and *in vivo* CP8 expression were used to challenge mice; five CC12 isolates (PFESA1194 (*in vitro* +, in vivo +), PFESA1305 (–, +), PFESA1405 (–, +), PFESA2455 (–, +), PFESA1502 (–,–)) and two CC188 isolates (PFESA2089 (+, not tested *in vivo*), PFESA2058 (–, +)). CD1 mice (7–9 weeks of age, 10 per group) were administered three IP injections of ~ 2x10^6^ CFU/animal in 0.5 mL at 0, 4 and 12 weeks and bled at week 27. CP8 antibody responses were measured using a CP8 specific cLIA assay. CP8 antibody responses were detected in mice infected with all isolates except the CC12-t160 isolate (PFESA1502) that didn’t express capsule in the IFA murine model.

Challenge CP8 Isolate	CP8 cLIA titer GMT (95% CI)^a^^,^^b^
PFESA1194	68.2 (6.3–735.7)
PFESA1305	17.3 (2.3–130)
PFESA1405	12.9 (1.3–123.2)
PFESA2455	10.4 (1.6–69.7)
PFESA1502	3 (3–3)
PFESA2089	5.7 (1.3–23.8)
PFESA2058	32.2 (2.1–499)

^a^ LOD values (units/mL) were 6.5 for CP5 and 3.0 for CP8. CP5 cLIA titer of all samples was 6.5.

^b^ A CP8 positive control was used in this experiment and it had CP8 cLIA GMT of 173.3 (95% CI: 14–2155).

## Discussion

Owing to the extended length of vaccine research and development, it is important to monitor the epidemiology of the target pathogen throughout the development program to ensure that the vaccine candidate under development remains appropriate. Over recent years, the US has seen a change in the epidemiology of *S*. *aureus* as exemplified by the rise of CA-MRSA USA300 isolates and their establishment in healthcare settings [9]. Analysis of these contemporary disease isolates revealed that they carried a mobile methicillin resistance cassette and specific virulence factors such as PVL, and appeared to lack capsular polysaccharides expression *in vitro* [55, 88, 90]. The most advanced vaccine candidate in clinical studies is SA4Ag, a four-antigen vaccine which includes CP5 and CP8 conjugated to the CRM197 carrier protein in addition to two protein antigens. The observation that emerging isolates may not express two of the vaccine’s target antigens, (CP5 or CP8) highlighted the importance to further investigate the distribution of capsule genotypes in prevalence-based studies and to assess the proportion of isolates that express capsular polysaccharides. This study is also the first that carefully and comprehensively examined the clonal relatedness (CC and *spa* types), CP genotype, and CP phenotype (*in vitro* and *in vivo*) amongst a panel of relevant *S*. *aureus* clinical isolates. A prevalence-based collection of such *S*. *aureus* isolates in the US was utilized to permit an epidemiological survey in a variety of clinical settings.

The *S*. *aureus* population circulating in the US during the time periods studied was represented by 13 prominent clonal complexes. In concordance with previous reports of *S*. *aureus* infection and colonization in the US and Europe, the *S*. *aureus* isolates in healthcare and community settings were primarily distributed between four major CC5, 8, 30, and 45 [91–96], with CC8 and CC5 comprising the largest number of isolates. In agreement with recent studies showing an association between invasive disease and clonality [97], we also observed a significant association between CC and type of infection (invasive vs non-invasive) (*P* = 0.036).

The inpatient data shows that although most *S*. *aureus* genotypes (CC) have the capacity to cause invasive disease, isolates within CC5, 8, 30 and 45 caused the majority of invasive disease. In the current study, the most prevalent *spa* types in the US *S*. *aureus* population were t008 and t002, consistent with results from earlier reports [14]. There was a strong association between *spa* type and type of infection (HA-wound vs respiratory or invasive) (*P* < 0.001), t008 isolates caused a higher proportion of wound infections whereas t002 isolates predominated in HA-invasive and HA-respiratory infections.

We determined CP prevalence, genotype distribution and expression in *S*. *aureus* isolates circulating in the US. Most available literature reports described capsule prevalence as determined by serotyping [53, 54, 56]. The present study is the first systematic molecular analysis of CP distribution and expression both *in vitro* and *in vivo* of *S*. *aureus* isolates causing disease in the US during 2004 and 2009–10. All *S*. *aureus* isolates harbored the genetic pathway to express either CP5 (72%) or CP8 (28%). A link was found between capsule genotype and CC-*spa* type, supporting the previous notion of the high clonality of the capsule antigens [67]. Among the MRSA isolates, 96% of the isolates were CP5 while 50% of the MSSA strains were CP5 (*P* < 0.001). Overall (MRSA and MSSA isolates combined), CP5 genotypes were the most prevalent in both inpatient and outpatient wards (*P* < 0.001).

For a vaccine strategy to be effective, the vaccine must contain antigens that are expressed in host microenvironments and elicit immune responses in humans. A mechanism of protection for multicomponent vaccines containing CP conjugates is to induce antibodies that facilitate the killing of the bacteria by complement mediated opsonophagocytosis which can be measured *in vitro* by OPA. We demonstrated in this study that humans immunized with a vaccine (SA3Ag) containing CP conjugates generated CP-specific antibodies with bactericidal activity against encapsulated isolates in OPA and that genetic lineage is not linked to the potential for a strain to be killed by anti-CP antibodies. CP-specific antibodies were detected in mice infected with two *S*. *aureus* USA300 isolates and not in mice infected with a USA300 capsule knockout mutant, indicating *in vivo* expression of capsule in these isolates (Table 5).

Potential vaccine candidate antigens must be present in the genome of circulating isolates, highly conserved, and expressed by *S*. *aureus* during the course of natural infection. The danger in relying on *in vitro* assays to preselect antigens is the potential lack of expression using standard culture conditions. Several studies comparing *S*. *aureus* gene expression *in vivo* to that under *in vitro* growth conditions indicated that the behavior of *S*. *aureus in vivo* could be significantly different from that observed *in vitro* [98–101]. Some candidate antigens are expressed both *in vivo* and *in vitro* (e.g. clumping factor A [21, 102, 103]), while others are poorly expressed or not detectable in *in vitro* grown bacteria [59, 104, 105]. Manganese transporter protein C (MntC), a protein that is not readily detected using standard culture conditions, was identified as a potential vaccine target based on *in vivo* expression analysis showing that MntC is rapidly upregulated by *S*. *aureus* in a murine model of infection [106]. In this study, assessing *S*. *aureus* isolates for capsule production revealed that a proportion of isolates tested within CC8 (37%) did not express capsule *in vitro*. Likewise, a lack of *in vitro* capsule expression was observed for other isolates belonging to CC5, 12, 97 and 188, albeit to a lesser extent. *In vivo* capsule expression analysis of representative isolates that showed no capsule expression *in vitro* revealed that many of these isolates were perfectly capable of expressing capsule *in vivo*. We therefore examined the *cap* operons of all isolates in the collection to identify whether any mutations within the *cap* operons could be linked to an *in vitro* CP-negative phenotype.

Both Cocchiaro et al. and Boyle-Vavra et al. [51, 55] identified specific genetic mutations in the capsular polysaccharide biosynthetic operon, which they showed by complementation experiments may contribute to the lack of observed capsule expression *in vitro*. In an effort to assess the relevance of these mutations with regard to capsule expression during infection, Boyle-Vavra et al. [55] conducted an *in vivo* analysis using a single isolate and confirmed that no capsule expression could be detected in that instance. The methodology used was different from that utilized in our previous reports where expression was detected [21, 64] so it is difficult to interpret these findings in relation to previous observations. The mutations in the *cap5A* promoter and the *cap5D*, *cap5E* and *cap5G* coding regions [55, 107] were also identified in CC8 isolates tested in this study (Table 3). We propose that only one of these mutations (type 2, *cap5D*) is likely to contribute to the lack of *in vitro* expression phenotype. The *cap5D* mutation is a frameshift mutation predicted to result in a truncated non-functional enzyme due to premature termination of protein synthesis. As the *cap5A* promoter mutation (mutation type 1) was identified in some strains that expressed capsule *in vitro*, this is unlikely to be the root cause of the acapsular phenotype. The *cap5E* (mutation type 3) and *cap5G* (mutation type 4) SNPs result in non-conservative amino acid substitutions (Cap5E D75Y and Cap5G F160L) with the potential to influence the activity of the corresponding enzymes. However, Cap5E D75Y maps to a location peripheral to cofactor or substrate binding sites [108], and Cap5G F160L falls beyond the boundary of gene fragments capable of genetically complementing the USA300 acapsular phenotype [55]. Additional mutations associated with other CC including CC5 (*cap5E*); CC12 (*cap8D* and *cap8E*) and CC188 (*cap8O*) were also identified in this study. In each of the cases tested, there was no correlation between these mutations and the ability of an isolate to express capsule *in vivo*. This was the case, even in the instance of the *cap8D* frame shift mutation in the two CC12 isolates (mutation type 6, Table 3), a mutation that might be expected to ablate expression of capsule functions. The third gene where a missense mutation was identified was *cap8O* in a CC188 isolate. This gene has been demonstrated to be essential for the production of D-ManNAcA when it is completely knocked out, however the effects of specific non-synonymous substitutions have not been characterized. These observations suggest that point/frameshift mutations within the capsular genetic pathway are generally not predictive of the ability of this pathogen to produce capsule during infection. The only clear capsule null mutation was identified in a single CC97 isolate within the collection of 516 *S*. *aureus* isolates, for which no detectable capsule expression was observed *in vitro*, suggesting that the acquisition of such mutations is potentially detrimental to *S*. *aureus* and thus clonal expansion of such isolates in the clinical setting may be unlikely. CC97 is an interesting lineage that is a leading cause of bovine mastitis globally [109, 110] and has several major genomic differences to the common *S*. *aureus* CC that cause disease in humans. The loss of capsule function in the CC97 isolate is consistent with the prevalence of acapsular bovine *S*. *aureus* that has evolved through mutations in capsular polysaccharide genes [51, 111].

Given our data, it seems possible that particular capsular biosynthetic enzymes that are not expressed or are inactive *in vitro* may be expressed or active in the *in vivo* environment; therefore *in vitro* analyses may not necessarily predict capsule expression *in vivo*. Rozemeijer et al. [112] reported the detection of RNA transcripts of genes encoding capsular polysaccharide in swab samples from infected patients by qRT-PCR despite the experimental challenges. The *in vivo* mouse experiments presented here have the disadvantage that the number of bacterial isolates that are recoverable is limited, making it difficult to assess the absolute structure of the polysaccharide being detected. Despite these limitations, sufficient bacterial RNA was isolated from mouse blood to confirm that individual gene transcripts of the *cap* operon were being expressed (Fig 4B).

We acknowledge that we have generated a body of data that can be considered controversial. Using specific capsular polysaccharide detection reagents and assays we show that clear differences exist between the expression of capsular polysaccharides by *S*. *aureus* disease-causing strains *in vivo*, compared with expression in the same strains grown in laboratory medium. At this point we can only speculate on possible mechanisms. Ouyang et al. [107] were the first to postulate that *in vitro* expression may be compromised due to a mutation within the *cap5A* promoter region. In this study we have found that this promoter mutation is not limited only to CP5 *S*. *aureus* strains that do not express capsule *in vitro*, it is also present in other strains where capsular polysaccharide expression is detected *in vitro*. A potential explanation for this was outlined by Sau et al. [113] who demonstrated that while the *cap* operon was transcribed as a single transcript using the *cap5A* associated promoter, weaker promoters were also associated with the individual genes. Likewise Ouyang et al. [107] and Gupta et al. [114] have demonstrated that the principal promoter (P*cap*) of the *cap* operon has several different activators and repressors that bind to different promotor sites. Genetic and biochemical data may explain how *cap5D* frameshift mutations associated with USA300 strains can be bypassed *in vivo*. Our transcriptional analyses demonstrates the presence of *cap5D* transcript during *in vivo* growth, suggesting that although the translation of the *cap5D* coding sequence may be disrupted by a frameshift, it is possible that a post-translational bypass mechanism may be responsible for compensating for the *cap5D* nucleotide insertion under *in vivo* growth conditions. The gene products of *capD* and *capE* are similar, with the exception of a membrane bound motif associated with *capD*, which is thought to anchor the polysaccharide to the cell surface during synthesis [115]. Both proteins encode UDP-GlcNAc C6 dehydratases that are associated with the first steps in D-FucNAc and L-FucNAc biosynthesis, respectively. However, Miyafusa et al. [108] demonstrated that the Cap5E enzyme is capable of producing the stereoisomeric precursors of both D-FucNAc and L-FucNAc *in vitro*. Not only can Cap5E generate the UDP-*xylo*-sugar (L-FucNAc precursor), but it can also produce the UDP-*arabino*-sugar (D-FucNAc precursor) as byproduct. The activity of the enzyme is also likely to be regulated by exogenous metabolites due to the presence of an allosteric site identified in the crystal structure [108]. By generating both D- and L- precursors of fucose, Cap5E has the potential to bypass Cap5D, thus providing a potential mechanism for our observations which could be explored further (S6 Fig).

In conclusion, our comprehensive study of relevant *S*. *aureus* disease isolates demonstrates that although the expression of capsule may not be fully understood *in vivo*, a much higher proportion of the disease-causing isolates in the US have the potential to express capsule *in vivo* than previously predicted through analysis of the pathogen cultured in *in vitro* growth media. Studies have identified many genes and pathways involved in capsule production by *S*. *aureus*, but the role and mechanisms by which they regulate capsule production during infection remain to be elucidated. Our work shows that mutations in the *cap* operon are identified in a subset of the 16 *cap* genes and they are associated with specific CC. Although all USA300 and some USA500 isolates within the CC8 lineage had the *cap5D* frameshift mutation, many were able to express capsule *in vivo*. Phylogenetic analysis of USA300 and USA500 isolates in the context of a wider CC8 population revealed that these two epidemic clones are not directly related [89], in contrast to a previous suggestion that USA500 represents a progenitor of the USA300 clone [116]. In this study, less than 10% of invasive disease cases were caused by USA300 isolates, where reduced expression of CP *in vitro* was observed. Therefore, surveys of CP prevalence that only take into account the *in vitro* phenotype or use isolate collections that may not represent the target population for the vaccine, will underestimate the distribution of CP-expressing isolates and thus the potential coverage of CP conjugate containing vaccines. The expression of capsule by *S*. *aureus* is only one of several virulence mechanisms deployed by this pathogen. It is expected that for a vaccine to be effective it will need to also address additional virulence mechanisms such as binding to host structures and an ability to scavenge nutrients [33, 106, 112, 117–119]. The appropriate clinical efficacy trials are therefore crucial to demonstrate the effectiveness of a multi-antigen vaccine containing capsular polysaccharide conjugates in preventing *S*. *aureus* infections.

## Supporting information

S1 Table Genotypic characteristics of *S*. *aureus* isolates. (PDF)

S2 Table Frequencies of *spa* types (excluding singletons and unknown *spa* types, *n* = 93) and associated clonal complexes (CC) and sequence types (ST). (PDF)

S3 Table Genotypic characteristics of *S*. *aureus* strains in the challenge stocks and after recovery from infected mice (two mice per strain) in the IFA experiments on *in vivo* derived bacteria. Presence of the four conserved *cap5* operon mutations is indicated in green while the absence of mutations is indicated in red.
(PDF)

S1 Fig Distribution of clonal complexes (CC) of clinical *S*. *aureus* isolates collected at different census regions in the US. (TIF)

S2 Fig Distribution of *spa* types among different healthcare and community-associated infections. (A) All isolates associated with *S*. *aureus* infections in healthcare and community settings. (B) *S*. *aureus* isolates associated with the main types of clinical infections in healthcare settings.
(DOCX)

S3 Fig Distribution of clonal complexes (CC) and capsular polysaccharide genotypes (CP5/8) of MRSA and MSSA isolates by year and ward source. (DOCX)

S4 Fig Approximately-maximum-likelihood phylogenetic trees of analyzed *S*. *aureus* CP5 (A and C) and CP8 (B and D) isolates annotated with the distribution of *cap* operon SNPs (A and B) and indels in US *S*. *aureus* isolates (C and D). (A) *cap* operon SNPs in CP5 isolates. SNPs were identified across the *cap* operon based on mapping against *S*. *aureus* HO 5096 0412 reference. Isolate tracks color-coded by CC (legend below figure). Color-coding of SNPs; Green A, Blue G, Black T and Red C. (B) *cap* operon SNPs in CP8 isolates. SNPs were identified across the *cap* operon and plotted against tree of all CP8 USA isolates based on mapping against *S*. *aureus* MSSA476 reference. Isolate tracks color-coded by CC legend below figure). (C) *cap* operon indels in CP5 isolates. Indels were identified across the cap operon and plotted against tree of all CP5 USA isolates based on mapping against *S*. *aureus* HO 5096 0412 reference. Isolate tracks color-coded by CC (legend below figure). Color-coding of indels; Vertical magenta dots indicates insertion while dark gray indicates deletion. For each isolate, the mapping coverage is indicated by the light grey horizontal field, with white regions demonstrating deletion of the corresponding *cap* genomic region. As such, this figure shows the partial loss of *cap5D-E* in a single CC97 isolate. (D) *cap* operon indels in CP8 isolates. Indels were identified across the *cap* operon and plotted against tree of all CP8 USA isolates based on mapping against *S*. *aureus* MSSA476 reference.
(DOCX)

S5 Fig Specificity of immunofluorescence assay (IFA) for the detection of surface capsular polysaccharides in the murine bacteremia model. Three different *S*. *aureus* strains: Reynolds (CP5), its isogenic CP-negative mutant, and the CP- negative Reynolds complemented with CP8-coding sequences were used to challenge mice. The strains were tested in a blinded fashion with two independent experiments per strain. *S*. *aureus* was collected at the time of challenge (T0) and from the blood of infected mice 6 hr post infection (T6), and stained with rabbit anti-CP5, or rabbit anti-CP8 antibodies, or normal rabbit IgGs. Bright-field (A) and fluorescence photographs (B) of IFA staining are shown for each strain at both time points.
(TIF)

S6 Fig Proposed Cap5D bypass mechanism. (A) Capsular polysaccharide type 5 is composed of repeat units of D-N-acetylmannosamine (D-ManNAc); L-N-acetylfucosamine (L-FucNAc) and D-L-acetylfucosamine (D-FucNAc). Cap5D is associated with synthesis of the D-FucNAc precursor (Li et al. Internatl. J. Med. Micro. 2014) and Cap5E is primarily associated with synthesizing L-FucNAc precursor (Miyafusa et al. FEBS Lett. 2013). (B) Cap5D is a 4, 6-dehydratase that converts UDP-D-GlcNAc to a D-FucNAc precursor, while Cap5E has 4, 6-dehydratase and 5-epimerase activity that converts UDP-D-GlcNAc to an L-FucNAc precursor but can also generate the analogous D-FucNAc precursor in a reverse epimerization reaction (Miyafusa et al. FEBS Lett. 2013). We propose that for *S*. *aureus* USA300 strains where the *cap5D* gene has a premature stop codon, the UDP-D-FucNAc precursor UDP-2-acetoamino-2, 6-dideoxy-a-D-xylo-4-hexulose (highlighted in orange) is derived as byproduct of the CapE epimerase reaction.
(DOCX)
