# Supplementary material for: Molecular epidemiology and expression of capsular polysaccharides in Staphylococcus aureus clinical isolates in the United States
Source: PLoS One. 2019 Jan 14;14(1):e0208356. doi: 10.1371/journal.pone.0208356 (PMC6331205; doi:10.1371/journal.pone.0208356)
Supplement: S1 Table — (PDF) [file pone.0208356.s001.pdf]

Isolate ID	ST	*spa* type	ENA accession
PFESA1191	5	t062	ERS411121
PFESA1192	45	t1946	ERS411129
PFESA1193	45	NT	ERS411137
PFESA1194	1156	t156	ERS411145
PFESA1195	5	t002	ERS411153
PFESA1196	5	t002	ERS411161
PFESA1197	30	t012	ERS411169
PFESA1198	85	t062	ERS411177
PFESA1199	3004	t062	ERS411185
PFESA1200	45	t553	ERS411193
PFESA1201	45	t004	ERS411201
PFESA1202	5	t062	ERS411209
PFESA1203	45	t004	ERS411073
PFESA1204	36	t018	ERS411122
PFESA1205	45	t671	ERS411130
PFESA1206	5	t002	ERS411138
PFESA1207	45	t004	ERS411146
PFESA1208	5	t062	ERS411154
PFESA1209	5	t002	ERS411162
PFESA1210	5	t002	ERS411170
PFESA1211	5	t002	ERS411178
PFESA1212	5	t002	ERS411186
PFESA1214	5	t002	ERS411194
PFESA1215	5	t002	ERS411202
PFESA1302	225	t045	ERS411210
PFESA1303	15	t346	ERS411123
PFESA1304	8	t024	ERS411131
PFESA1305	12	t5318	ERS411139
PFESA1306	8	t008	ERS411147
PFESA1307	3005	t088	ERS411155
PFESA1308	8	t008	ERS411163
PFESA1309	8	t008	ERS411171
PFESA1311	8	t008	ERS411179
PFESA1312	8	t008	ERS411187
PFESA1313	5	t002	ERS411195
PFESA1314	30	t012	ERS411203
PFESA1315	3016	t040	ERS411211
PFESA1316	5	t002	ERS411124
PFESA1317	8	t008	ERS411132
PFESA1318	8	t064	ERS411140

Isolate ID	ST	*spa* type	ENA accession
PFESA1319	225	t045	ERS411148
PFESA1320	225	t045	ERS411156
PFESA1321	225	t045	ERS411164
PFESA1322	8	t008	ERS411172
PFESA1323	30	t021	ERS411180
PFESA1324	97	t267	ERS411188
PFESA1325	225	t045	ERS411196
PFESA1326	8	t008	ERS411112
PFESA1327	8	t068	ERS411120
PFESA1328	8	t008	ERS411204
PFESA1329	8	t008	ERS410929
PFESA1330	72	t1346	ERS410937
PFESA1331	45	t061	ERS411212
PFESA1332	15	t279	ERS411125
PFESA1333	8	t008	ERS411133
PFESA1334	45	t908	ERS410945
PFESA1335	8	t008	ERS411213
PFESA1337	8	t024	ERS410961
PFESA1338	5	t002	ERS410969
PFESA1339	8	t008	ERS411205
PFESA1340	188	t189	ERS411197
PFESA1341	8	t008	ERS410977
PFESA1342	8	t008	ERS410985
PFESA1343	45	t061	ERS410993
PFESA1344	8	NT	ERS411001
PFESA1345	8	t008	ERS411009
PFESA1346	8	t024	ERS411189
PFESA1347	8	t008	ERS411017
PFESA1348	15	t279	ERS411181
PFESA1349	45	t908	ERS410930
PFESA1350	8	t008	ERS411173
PFESA1351	8	t064	ERS410938
PFESA1352	8	t008	ERS410946
PFESA1353	8	t008	ERS410954
PFESA1354	8	t008	ERS410962
PFESA1355	8	t008	ERS410970
PFESA1357	8	t008	ERS410986
PFESA1358	8	t008	ERS410994
PFESA1359	8	t008	ERS411002
PFESA1360	8	t008	ERS411010

Isolate ID	ST	*spa* type	ENA accession
PFESA1361	8	t064	ERS411165
PFESA1362	8	t008	ERS411018
PFESA1363	109	t209	ERS410931
PFESA1364	8	t008	ERS410939
PFESA1365	8	t008	ERS411157
PFESA1366	8	t008	ERS411149
PFESA1367	8	t121	ERS410947
PFESA1368	8	t008	ERS411141
PFESA1369	8	t008	ERS410955
PFESA1370	8	t4166	ERS410963
PFESA1371	8	t008	ERS411126
PFESA1387	88	t786	ERS411134
PFESA1388	8	t024	ERS410996
PFESA1389	5	t002	ERS411004
PFESA1390	8	t2558	ERS411142
PFESA1391	8	t008	ERS411150
PFESA1392	105	t002	ERS411158
PFESA1393	144	t10017	ERS411166
PFESA1394	5	t002	ERS411174
PFESA1395	5	t214	ERS411182
PFESA1396	50	t8818	ERS411012
PFESA1397	5	t002	ERS411190
PFESA1398	8	t008	ERS411020
PFESA1399	8	t008	ERS410933
PFESA1400	5	t4371	ERS411198
PFESA1401	30	t012	ERS411206
PFESA1402	2562	t1265	ERS410941
PFESA1403	5	t4863	ERS411214
PFESA1404	8	t008	ERS410949
PFESA1405	12	t771	ERS411127
PFESA1406	5	t002	ERS411135
PFESA1407	5	t306	ERS411143
PFESA1408	87	t216	ERS411151
PFESA1409	30	t021	ERS411159
PFESA1410	8	t008	ERS411167
PFESA1411	5	t002	ERS411175
PFESA1450	2997	t3736	ERS410976
PFESA1451	2996	t012	ERS410984
PFESA1476	8	t008	ERS410890
PFESA1477	8	t008	ERS410898

Isolate ID	ST	*spa* type	ENA accession
PFESA1478	8	t334	ERS410906
PFESA1479	15	t084	ERS410914
PFESA1480	105	t002	ERS410922
PFESA1481	231	t002	ERS410835
PFESA1482	45	t277	ERS410843
PFESA1483	15	t547	ERS410851
PFESA1484	25	t078	ERS410859
PFESA1485	8	t008	ERS410867
PFESA1486	22	t005	ERS410875
PFESA1487	9	t193	ERS410883
PFESA1488	582	t084	ERS410891
PFESA1489	8	t008	ERS410899
PFESA1490	8	t008	ERS410907
PFESA1491	8	t008	ERS410915
PFESA1492	8	t008	ERS410923
PFESA1493	5	t688	ERS410836
PFESA1494	45	t630	ERS410844
PFESA1495	1	t2192	ERS410852
PFESA1496	25	t078	ERS410860
PFESA1497	8	t008	ERS410868
PFESA1498	8	t008	ERS410876
PFESA1499	109	t209	ERS410884
PFESA1500	25	t7048	ERS410892
PFESA1501	231	t002	ERS410900
PFESA1502	12	t160	ERS410908
PFESA1528	88	t692	ERS410926
PFESA1529	105	t002	ERS410839
PFESA1530	30	t743	ERS410847
PFESA1531	231	t002	ERS410855
PFESA1532	8	t008	ERS410863
PFESA1533	88	t692	ERS410871
PFESA1534	5	t002	ERS410879
PFESA1535	121	NT	ERS410887
PFESA1536	30	t021	ERS410895
PFESA1537	8	t008	ERS410903
PFESA1538	8	t008	ERS410911
PFESA1539	59	t216	ERS410919
PFESA1540	5	t002	ERS410927
PFESA1541	8	t008	ERS410840
PFESA1542	3015	t008	ERS410848

Isolate ID	ST	*spa* type	ENA accession
PFESA1543	3003	t088	ERS410856
PFESA1544	105	t002	ERS410864
PFESA1545	8	t008	ERS410872
PFESA1546	30	t275	ERS410880
PFESA1547	8	t008	ERS410888
PFESA1548	2253	t008	ERS410896
PFESA1549	6	t701	ERS410904
PFESA1550	5	t002	ERS410912
PFESA1551	15	t346	ERS410920
PFESA1577	3014	t1062	ERS410739
PFESA1578	5	t045	ERS411183
PFESA1579	8	t008	ERS410747
PFESA1580	8	t008	ERS410755
PFESA1581	5	t002	ERS411191
PFESA1582	59	t8419	ERS410763
PFESA1583	8	t008	ERS410771
PFESA1584	8	t008	ERS410779
PFESA1585	8	t008	ERS411199
PFESA1586	8	t008	ERS410787
PFESA1587	508	t2444	ERS410795
PFESA1588	25	t258	ERS410803
PFESA1589	8	t008	ERS410811
PFESA1590	8	t008	ERS411207
PFESA1591	8	t008	ERS410819
PFESA1592	97	t2734	ERS410827
PFESA1593	8	t008	ERS410740
PFESA1594	8	t008	ERS410748
PFESA1595	5	t002	ERS411215
PFESA1596	15	t385	ERS411128
PFESA1597	5	t548	ERS411136
PFESA1598	8	t024	ERS410756
PFESA1599	5	t002	ERS411144
PFESA1600	5	t1265	ERS411152
PFESA1601	5	t002	ERS411160
PFESA1602	15	NT	ERS410764
PFESA1603	5	t002	ERS410772
PFESA1604	5	t1228	ERS410780
PFESA1605	30	t018	ERS410788
PFESA1606	8	t008	ERS410796
PFESA1607	2995	t012	ERS410804

Isolate ID	ST	*spa* type	ENA accession
PFESA1608	496	t002	ERS410812
PFESA1609	105	t002	ERS410820
PFESA1610	97	t267	ERS410828
PFESA1611	30	t338	ERS410741
PFESA1612	88	t730	ERS410749
PFESA1613	5	t002	ERS410757
PFESA1614	8	t008	ERS410765
PFESA1615	50	t185	ERS410773
PFESA1616	2999	t002	ERS410781
PFESA1617	3000	t008	ERS410789
PFESA1618	8	t008	ERS410797
PFESA1619	5	t002	ERS410805
PFESA1776	45	NT	ERS410715
PFESA1777	5	t002	ERS410723
PFESA1778	5	t002	ERS410731
PFESA1779	45	t644	ERS410644
PFESA1780	45	t644	ERS410652
PFESA1781	20	t731	ERS410660
PFESA1782	109	t209	ERS410668
PFESA1783	5	t071	ERS410676
PFESA1784	5	t002	ERS410684
PFESA1785	8	t008	ERS410692
PFESA1786	5	t179	ERS410700
PFESA1787	8	t008	ERS410708
PFESA1788	8	t008	ERS410716
PFESA1789	45	t4460	ERS410724
PFESA1790	5	t002	ERS410732
PFESA1791	8	t008	ERS410645
PFESA1792	3006	t002	ERS410653
PFESA1793	30	t399	ERS410661
PFESA1794	5	t002	ERS410669
PFESA1795	8	t064	ERS410677
PFESA1796	45	t553	ERS410685
PFESA1797	30	t2018	ERS410693
PFESA1798	5	t002	ERS410701
PFESA1799	30	t012	ERS410709
PFESA1800	5	t062	ERS410717
PFESA1801	97	t359	ERS410725
PFESA1802	105	t002	ERS410733
PFESA1803	5	t002	ERS410646

Isolate ID	ST	*spa* type	ENA accession
PFESA1804	256	t1964	ERS410654
PFESA1805	5	t002	ERS410662
PFESA1806	30	t3732	ERS410670
PFESA1807	105	t002	ERS410678
PFESA1808	25	t078	ERS410686
PFESA1878	87	t216	ERS410573
PFESA1879	1	t174	ERS410581
PFESA1880	8	t008	ERS410589
PFESA1881	109	t209	ERS410597
PFESA1882	8	t008	ERS410605
PFESA1883	8	t008	ERS410613
PFESA1884	8	t024	ERS410621
PFESA1885	8	t008	ERS410629
PFESA1886	51	NT	ERS410637
PFESA1887	87	t216	ERS410550
PFESA1888	8	t008	ERS410558
PFESA1889	8	t008	ERS410566
PFESA1890	8	t622	ERS410574
PFESA1891	188	t189	ERS410582
PFESA1892	36	t012	ERS410590
PFESA1893	45	t015	ERS410598
PFESA1894	5	t002	ERS410606
PFESA1895	105	t002	ERS410614
PFESA1896	188	t189	ERS410622
PFESA1897	2276	NT	ERS410630
PFESA1898	5	t002	ERS410638
PFESA1972	3002	t3169	ERS432037
PFESA1973	3017	t050	ERS432045
PFESA1974	8	t064	ERS411168
PFESA1975	8	t008	ERS411176
PFESA1976	8	t008	ERS411184
PFESA1977	8	t008	ERS432053
PFESA1978	8	t008	ERS432061
PFESA1979	72	t148	ERS432069
PFESA1980	8	t064	ERS432077
PFESA1981	8	t681	ERS411192
PFESA1982	8	t622	ERS432085
PFESA1983	3008	t008	ERS432093
PFESA1984	8	t064	ERS411200
PFESA1985	8	t064	ERS411208

Isolate ID	ST	*spa* type	ENA accession
PFESA1987	8	t064	ERS365739
PFESA1988	8	NT	ERS365747
PFESA1989	8	t1705	ERS365755
PFESA1990	8	t681	ERS365763
PFESA2027	59	t216	ERS432104
PFESA2028	105	t002	ERS432112
PFESA2029	5	t002	ERS432120
PFESA2030	15	t254	ERS432033
PFESA2031	105	t002	ERS432041
PFESA2032	188	NT	ERS432049
PFESA2033	3009	t015	ERS432057
PFESA2034	8	t008	ERS432065
PFESA2035	45	t553	ERS432073
PFESA2036	5	t002	ERS432081
PFESA2037	105	t002	ERS432089
PFESA2038	30	t012	ERS432097
PFESA2053	45	t950	ERS365771
PFESA2054	3010	t008	ERS409812
PFESA2055	8	t008	ERS365779
PFESA2056	8	t008	ERS365787
PFESA2057	8	t008	ERS365795
PFESA2058	188	t189	ERS365803
PFESA2059	8	t008	ERS365811
PFESA2061	5	t002	ERS365819
PFESA2062	8	t008	ERS365827
PFESA2063	8	t008	ERS365740
PFESA2064	8	t008	ERS365748
PFESA2065	15	t084	ERS365756
PFESA2066	5	t002	ERS365764
PFESA2067	8	t008	ERS365772
PFESA2068	30	t012	ERS365780
PFESA2069	45	t026	ERS365788
PFESA2070	8	t008	ERS365796
PFESA2071	5	t1084	ERS365804
PFESA2072	5	t002	ERS365812
PFESA2073	2319	t008	ERS365820
PFESA2074	8	t008	ERS365828
PFESA2075	8	t008	ERS410365
PFESA2076	5	t002	ERS365741
PFESA2077	8	t008	ERS365749

Isolate ID	ST	*spa* type	ENA accession
PFESA2078	97	t267	ERS365757
PFESA2079	5	t002	ERS365765
PFESA2080	105	t002	ERS365773
PFESA2081	188	t189	ERS365781
PFESA2082	5	NT	ERS365789
PFESA2083	8	t008	ERS365797
PFESA2084	8	t008	ERS365805
PFESA2085	5	t002	ERS365813
PFESA2086	8	t064	ERS365821
PFESA2087	8	t1578	ERS365829
PFESA2088	5	t002	ERS365742
PFESA2089	188	t189	ERS365750
PFESA2090	8	t008	ERS365758
PFESA2091	8	t008	ERS365766
PFESA2092	5	t002	ERS365774
PFESA2093	5	t002	ERS365782
PFESA2094	8	t1882	ERS365790
PFESA2095	5	t242	ERS365798
PFESA2096	225	t045	ERS365806
PFESA2097	5	t002	ERS365814
PFESA2098	8	t008	ERS365822
PFESA2100	5	t002	ERS365743
PFESA2101	225	t045	ERS365751
PFESA2102	97	t267	ERS365759
PFESA2103	8	t008	ERS365767
PFESA2104	8	t008	ERS365775
PFESA2153	8	t008	ERS410377
PFESA2154	5	t002	ERS365783
PFESA2155	5	t002	ERS410385
PFESA2156	8	t024	ERS410393
PFESA2157	8	t008	ERS365791
PFESA2158	8	t008	ERS410401
PFESA2159	30	t338	ERS365799
PFESA2160	2374	t021	ERS365807
PFESA2161	8	t008	ERS365815
PFESA2162	1866	t179	ERS365823
PFESA2163	8	t008	ERS410409
PFESA2164	8	t008	ERS365831
PFESA2165	5	t002	ERS365744
PFESA2166	8	t008	ERS410417

Isolate ID	ST	*spa* type	ENA accession
PFESA2167	5	t1303	ERS410425
PFESA2168	34	NT	ERS410433
PFESA2169	8	t008	ERS410441
PFESA2170	8	t211	ERS410449
PFESA2171	5	t1303	ERS410362
PFESA2172	25	t081	ERS410370
PFESA2173	5	t002	ERS365752
PFESA2174	87	t216	ERS365760
PFESA2175	8	t008	ERS410378
PFESA2176	8	t024	ERS410386
PFESA2177	5	t002	ERS410394
PFESA2178	8	t008	ERS410402
PFESA2179	5	t002	ERS410410
PFESA2180	8	t008	ERS410418
PFESA2181	5	t002	ERS410426
PFESA2182	105	t002	ERS410434
PFESA2183	5	t002	ERS410442
PFESA2184	8	t008	ERS410450
PFESA2185	121	NT	ERS410363
PFESA2186	72	t1991	ERS410371
PFESA2187	8	t2179	ERS410379
PFESA2188	8	t008	ERS410387
PFESA2189	2253	t008	ERS410395
PFESA2190	105	t002	ERS410403
PFESA2191	105	t002	ERS410411
PFESA2192	8	t008	ERS410419
PFESA2193	8	t008	ERS410427
PFESA2194	105	t002	ERS410435
PFESA2195	5	t002	ERS410443
PFESA2196	8	t008	ERS410451
PFESA2197	5	t002	ERS410364
PFESA2198	105	t002	ERS410372
PFESA2199	8	t064	ERS410380
PFESA2200	15	t084	ERS410388
PFESA2201	3011	t1315	ERS410396
PFESA2202	5	t002	ERS410404
PFESA2203	2253	t008	ERS410412
PFESA2204	15	t10352	ERS410420
PFESA2205	59	t216	ERS410428
PFESA2206	5	t306	ERS410436

Isolate ID	ST	*spa* type	ENA accession
PFESA2267	256	NT	ERS365768
PFESA2268	45	t671	ERS410347
PFESA2269	59	t216	ERS410355
PFESA2270	3013	t094	ERS365776
PFESA2271	5	t668	ERS410268
PFESA2272	8	t008	ERS410276
PFESA2273	8	t008	ERS410284
PFESA2274	8	t008	ERS365784
PFESA2275	105	t002	ERS365792
PFESA2276	109	t209	ERS365800
PFESA2277	8	t1348	ERS365808
PFESA2278	3007	t008	ERS410292
PFESA2279	1150	NT	ERS365816
PFESA2280	8	t334	ERS365824
PFESA2281	8	t121	ERS234133
PFESA2282	8	t008	ERS365745
PFESA2283	8	t008	ERS410300
PFESA2284	97	t2802	ERS365753
PFESA2285	8	t008	ERS410308
PFESA2286	105	t002	ERS410316
PFESA2287	15	t084	ERS365761
PFESA2288	5	t002	ERS365769
PFESA2289	5	t688	ERS365777
PFESA2290	2994	t012	ERS365785
PFESA2291	1003	t084	ERS365793
PFESA2292	5	t002	ERS365801
PFESA2293	8	t008	ERS365809
PFESA2294	105	t002	ERS365817
PFESA2295	5	t002	ERS365825
PFESA2296	5	1002	ERS234134
PFESA2297	5	t002	ERS365746
PFESA2298	8	t008	ERS365754
PFESA2299	30	t012	ERS365762
PFESA2300	5	t062	ERS365770
PFESA2301	45	t715	ERS365778
PFESA2302	25	t078	ERS365786
PFESA2303	8	t008	ERS365794
PFESA2304	30	t6997	ERS365802
PFESA2323	59	t216	ERS410270
PFESA2324	30	t318	ERS410278

Isolate ID	ST	*spa* type	ENA accession
PFESA2325	87	t216	ERS410286
PFESA2326	30	t019	ERS410294
PFESA2327	59	t216	ERS410302
PFESA2328	30	t018	ERS410310
PFESA2329	8	t008	ERS410318
PFESA2330	976	t008	ERS410326
PFESA2331	8	t3240	ERS410334
PFESA2332	8	t008	ERS410342
PFESA2333	5	t002	ERS409836
PFESA2410	15	t346	ERS365810
PFESA2411	72	t148	ERS410206
PFESA2412	59	t216	ERS365818
PFESA2413	8	t008	ERS365826
PFESA2414	2224	t185	ERS411025
PFESA2415	5	t002	ERS411033
PFESA2416	188	t189	ERS411041
PFESA2417	8	t008	ERS411049
PFESA2418	30	t338	ERS411057
PFESA2419	30	t338	ERS411065
PFESA2420	3012	t363	ERS410214
PFESA2421	45	t8814	ERS410222
PFESA2422	5	t002	ERS410230
PFESA2423	5	t002	ERS410238
PFESA2424	45	t644	ERS410246
PFESA2425	45	t644	ERS410254
PFESA2426	20	t731	ERS410262
PFESA2427	109	t209	ERS410175
PFESA2428	5	t071	ERS410183
PFESA2429	5	t002	ERS410191
PFESA2430	5	t179	ERS410199
PFESA2431	8	t008	ERS410207
PFESA2432	8	t008	ERS410215
PFESA2433	45	t4460	ERS410223
PFESA2434	5	t002	ERS410231
PFESA2435	8	t008	ERS410239
PFESA2436	3006	t002	ERS410247
PFESA2437	30	t399	ERS410255
PFESA2438	5	t002	ERS409844
PFESA2439	8	t064	ERS432122
PFESA2440	45	t553	ERS432130

Isolate ID	ST	*spa* type	ENA accession
PFESA2441	30	t2018	ERS432138
PFESA2442	5	t002	ERS432146
PFESA2443	30	t012	ERS432154
PFESA2444	5	t062	ERS432162
PFESA2445	59	t437	ERS432170
PFESA2447	1	t1178	ERS432186
PFESA2448	51	NT	ERS432194
PFESA2449	8	t008	ERS432202
PFESA2450	97	t2297	ERS432210
PFESA2451	5	t002	ERS432123
PFESA2452	2998	t1875	ERS432131
PFESA2453	8	t008	ERS432139
PFESA2454	30	t2485	ERS432147
PFESA2455	12	t160	ERS432155
PFESA2456	5	t306	ERS432163
PFESA2457	30	t1573	ERS432171
PFESA2458	30	t012	ERS432179
PFESA2459	30	t018	ERS432187
PFESA2460	8	t008	ERS432195
PFESA2461	5	t1154	ERS432203
PFESA2462	15	t085	ERS432211
PFESA2463	97	t3380	ERS432124
PFESA2464	39	t2387	ERS432132
PFESA2465	15	t254	ERS432140
PFESA2466	59	t216	ERS432148
PFESA2467	105	t002	ERS432156
PFESA2468	105	t002	ERS432164
PFESA2469	8	t024	ERS432172
PFESA2470	15	t605	ERS432180
PFESA2471	45	t821	ERS432188
PFESA2472	508	t050	ERS432196
PFESA2473	3001	t121	ERS432204
PFESA2474	5	t002	ERS432212
PFESA2742	8	t8286	ERS409790
PFESA2743	5	t002	ERS409798
PFESA2763	8	t681	ERS409806
