# Supplementary material for: Molecular epidemiology and expression of capsular polysaccharides in Staphylococcus aureus clinical isolates in the United States
Source: PLoS One. 2019 Jan 14;14(1):e0208356. doi: 10.1371/journal.pone.0208356 (PMC6331205; doi:10.1371/journal.pone.0208356)
Supplement: S2 Table — (PDF) [file pone.0208356.s002.pdf]

spa type	Number of isolates	Associated CC	Associated ST
t008	146	CC8	ST8, ST976, ST2253, ST2319, ST3000, ST3007, ST3008, ST3010, ST3015
t002	105	CC5	ST5, ST105, ST231, ST496, ST2999, ST3006
t012	13	CC30	ST30, ST36, ST2994, ST2995, ST2996
t216	13	CC59	ST59, ST87
t064	12	CC8	ST8
t024	9	CC8	ST8
t045	8	CC5	ST5, ST225
t062	8	CC5	ST5, ST85, ST3004
t189	7	CC188	ST188
t084	6	CC15	ST15, ST582, ST1003
t209	6	CC9	ST109
t018	4	CC30	ST30, ST36
t021	4	CC30	ST30, ST2374
t078	4	CC25	ST25
t267	4	CC97	ST97
t338	4	CC30	ST30
t553	4	CC45	ST45
t644	4	CC45	ST45
t004	3	CC45	ST45
t121	3	CC8	ST8, ST3001
t179	3	CC5	ST5, ST1866
t306	3	CC5	ST5
t346	3	CC15	ST15
t681	3	CC8	ST8

spa type	Number of isolates	Associated CC	Associated ST
t015	2	CC45	ST45, ST3009
t050	2	CC45	ST508, ST3017
t061	2	CC45	ST45
t071	2	CC5	ST5
t088	2	CC5	ST3003, ST3005
t1265	2	CC5	ST5, ST2562
t1303	2	CC5	ST5
t148	2	CC72	ST72
t160	2	CC12	ST12
t185	2	CC50	ST50, ST2224
t254	2	CC15	ST15
t279	2	CC15	ST15
t334	2	CC8	ST8
t399	2	CC30	ST
t622	2	CC8	ST8
t671	2	CC45	ST45
t688	2	CC5	ST5
t692	2	CC88	ST88
t731	2	CC20	ST20
t908	2	CC45	ST45
t2018	2	CC30	ST30
t4460	2	CC45	ST45
