# Supplementary material for: Molecular epidemiology and expression of capsular polysaccharides in Staphylococcus aureus clinical isolates in the United States
Source: PLoS One. 2019 Jan 14;14(1):e0208356. doi: 10.1371/journal.pone.0208356 (PMC6331205; doi:10.1371/journal.pone.0208356)
Supplement: S3 Table — Presence of the four conserved cap5 operon mutations is indicated in green while the absence of mutations is indicated in red. (PDF) [file pone.0208356.s003.pdf]

Bacterial strain_Colony number	ST	*spa* type	*cap5A* promoter mutation	*cap5D* frameshift insertion	*cap5E* mutation	*cap5G* mutation
**Reynolds strain**						
PFESA0266 Challenge_C1	25	t287				
PFESA0266 Challenge_C2	25	t287				
PFESA0266 Challenge_C3	25	t287				
PFESA0266 Challenge_C4	25	t287				
PFESA0266 Challenge_C5	25	t287				
PFESA0266 animal 5_C1	25	t287				
PFESA0266 animal 5_C2	25	t287				
PFESA0266 animal 5_C3	25	t287				
PFESA0266 Animal 5_C4	25	t287				
PFESA0266 Animal 5_C5	25	t287				
PFESA0266 Animal 6_C1	25	t287				
PFESA0266 Animal 6_C2	25	t287				
PFESA0266 Animal 6_C3	25	t287				
PFESA0266 Animal 6_C4	25	t287				
PFESA0266 Animal 6_C5	25	t287				
**PFESA0119**						
PFESA0119 Challenge_C1	8	t008				
PFESA0119 Challenge_C2	8	t008				
PFESA0119 Challenge_C3	8	t008				
PFESA0119 Challenge_C4	8	t008				
PFESA0119 Challenge_C5	8	t008				
PFESA0119 Animal1_C1	8	t008				
PFESA0119 Animal1_C2	8	t008				
PFESA0119 Animal1_C3	8	t008				
PFESA0119 Animal1_C4	8	t008				
PFESA0119 Animal1_C5	8	t008				
PFESA0119 Animal1_C6	8	t008				
PFESA0119 Animal1_C7	8	t008				
PFESA0119 Animal1_C8	8	t008				

Bacterial strain_Colony number	ST	*spa* type	*cap5A* promoter mutation	*cap5D* frameshift insertion	*cap5E* mutation	*cap5G* mutation
PFESA0119 Animal1_C9	8	t008				
PFESA0119 Animal1_C10	8	t008				
PFESA0119 Animal2_C1	8	t008				
PFESA0119 Animal2_C2	8	t008				
PFESA0119 Animal2_C3	8	t008				
PFESA0119 Animal2_C4	8	t008				
PFESA0119 Animal2_C5	8	t008				
PFESA0119 Animal2_C6	8	t008				
PFESA0119 Animal2_C7	8	t008				
PFESA0119 Animal2_C8	8	t008				
PFESA0119 Animal2_C9	8	t008				
PFESA0119 Animal2_C10	8	t008				
**PFESA0029**						
PFESA0029 Challenge_C1	8	t008				
PFESA0029 Challenge_C2	8	t008				
PFESA0029 Challenge_C3	8	t008				
PFESA0029 Challenge_C4	8	t008				
PFESA0029 Challenge_C5	8	t008				
PFESA0029 Animal3_C1	8	t008				
PFESA0029 Animal3_C2	8	t008				
PFESA0029 Animal3_C3	8	t008				
PFESA0029 Animal3_C4	8	t008				
PFESA0029 Animal3_C5	8	t008				
PFESA0029 Animal3_C6	8	t008				
PFESA0029 Animal3_C7	8	t008				
PFESA0029 Animal3_C8	8	t008				
PFESA0029 Animal3_C9	8	t008				
PFESA0029 Animal3_C10	8	t008				
PFESA0029 Animal4_C1	8	t008				
PFESA0029 Animal4_C2	8	t008				

Bacterial strain_Colony number	ST	*spa* type	*cap5A* promoter mutation	*cap5D* frameshift insertion	*cap5E* mutation	*cap5G* mutation
PFESA0029 Animal4_C3	8	t008				
PFESA0029 Animal4_C4	8	t008				
PFESA0029 Animal4_C5	8	t008				
PFESA0029 Animal4_C6	8	t008				
PFESA0029 Animal4_C7	8	t008				
PFESA0029 Animal4_C8	8	t008				
PFESA0029 Animal4_C9	8	t008				
PFESA0029 Animal4_C10	8	t008				
**PFESA0021**						
PFESA00021 Challenge_C1	8	t068				
PFESA00021 Challenge_C2	8	t068				
PFESA00021 Challenge_C3	8	t068				
PFESA00021 Challenge_C4	8	t068				
PFESA00021 Challenge_C5	8	t068				
PFESA00021 Animal7_C1	8	t068				
PFESA00021 Animal7_C2	8	t068				
PFESA00021 Animal7_C3	8	t068				
PFESA00021 Animal7_C4	8	t068				
PFESA00021 Animal7_C5	8	t068				
PFESA00021 Animal7_C6	8	t068				
PFESA00021 Animal7_C7	8	t068				
PFESA00021 Animal7_C8	8	t068				
PFESA00021 Animal7_C9	8	t068				
PFESA00021 Animal7_C10	8	t068				
PFESA00021 Animal8_C1	8	t068				
PFESA00021 Animal8_C2	8	t068				
PFESA00021 Animal8_C3	8	t068				
PFESA00021 Animal8_C4	8	t068				
PFESA00021 Animal8_C5	8	t068				
PFESA00021 Animal8_C6	8	t068				

**Bacterial strain_Colony number**	**ST**	***spa* type**	***cap5A* promoter mutation**	***cap5D* frameshift insertion**	***cap5E* mutation**	***cap5G* mutation**
PFESA00021 Animal8_C7	8	t068				
PFESA00021 Animal8_C8	8	t068				
PFESA00021 Animal8_C9	8	t068				
PFESA00021 Animal8_C10	8	t068				
