# Supplementary figures and images for: Molecular epidemiology and expression of capsular polysaccharides in Staphylococcus aureus clinical isolates in the United States
Source: PLoS One. 2019 Jan 14;14(1):e0208356. doi: 10.1371/journal.pone.0208356 (PMC6331205; doi:10.1371/journal.pone.0208356)

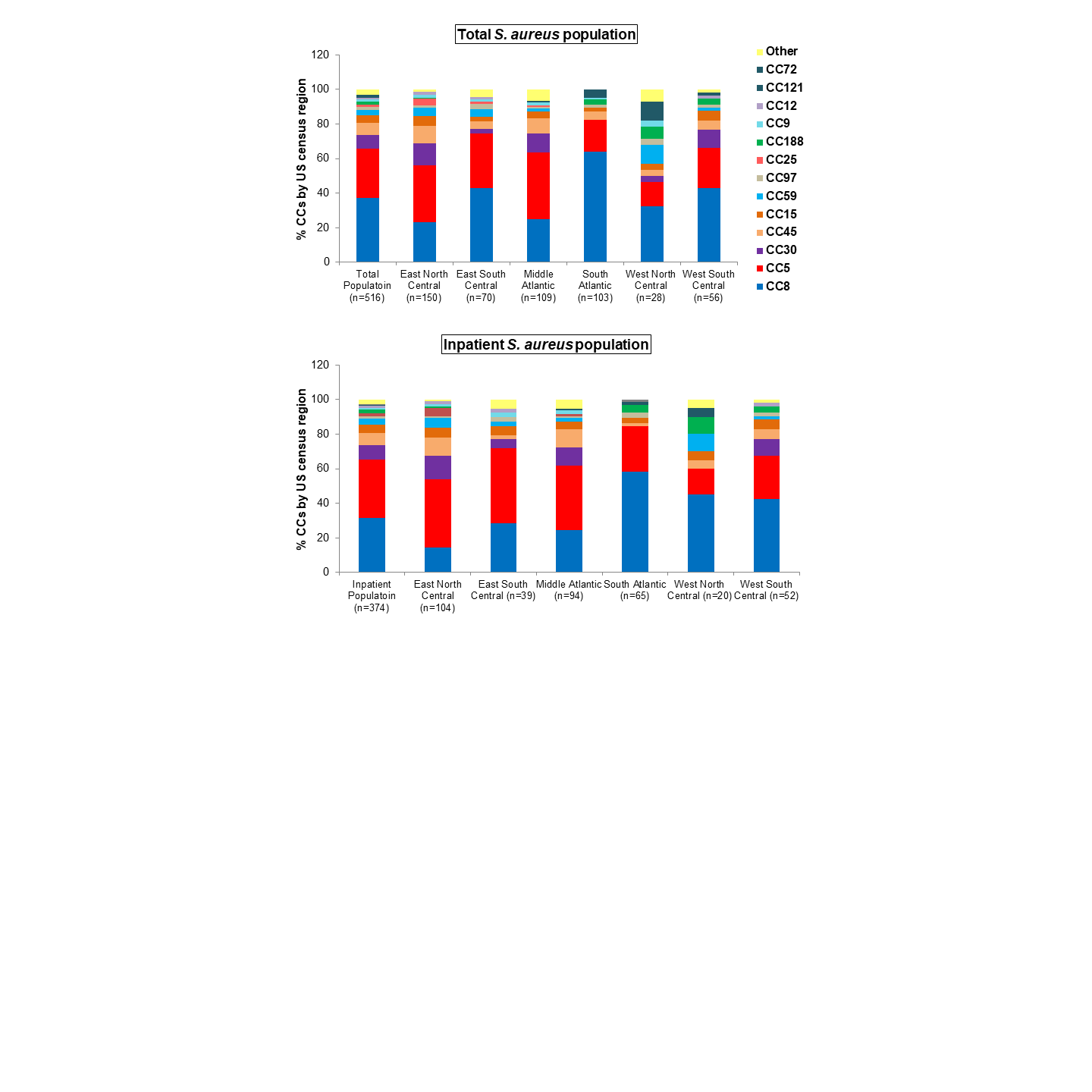

Supplement: S1 Fig — (TIF) [file pone.0208356.s004.tif]

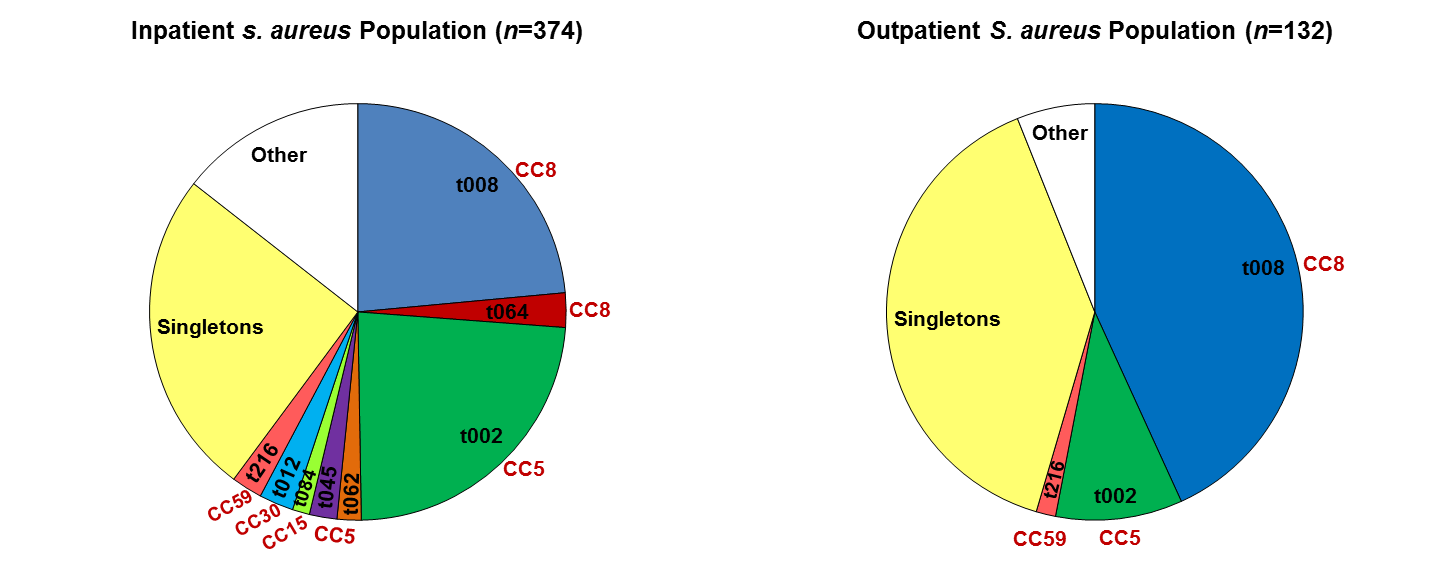


**A**

**B**


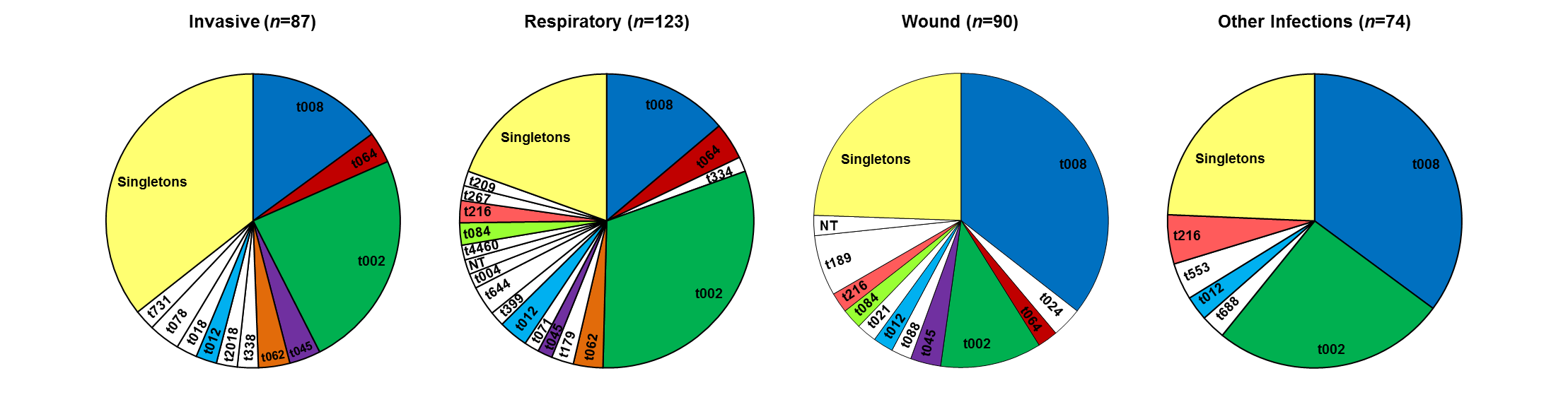

Supplement: S2 Fig — (A) All isolates associated with S. aureus infections in healthcare and community settings. (B) S. aureus isolates associated with the main types of clinical infections in healthcare settings. (DOCX) [file pone.0208356.s005.docx]

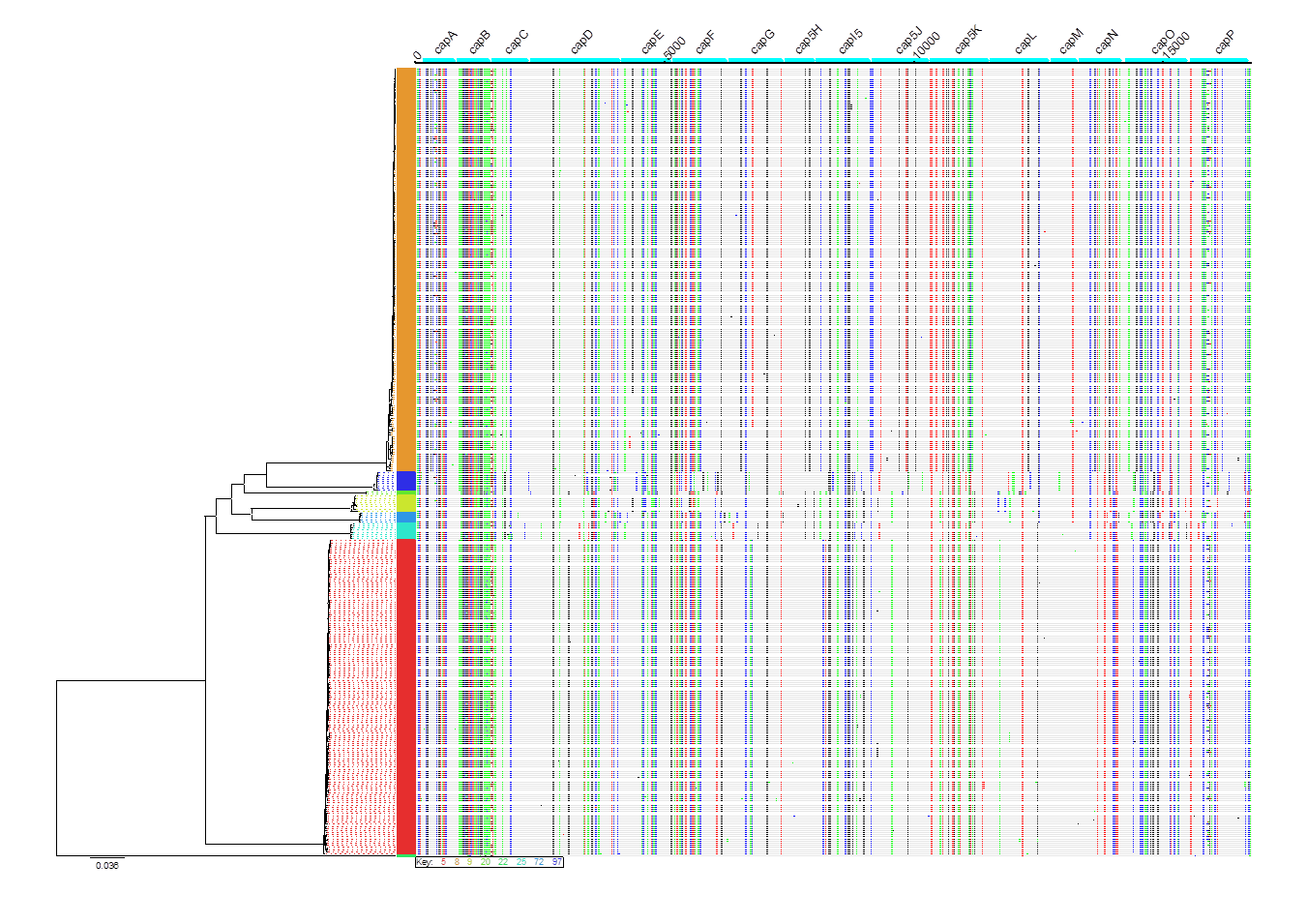


**A**


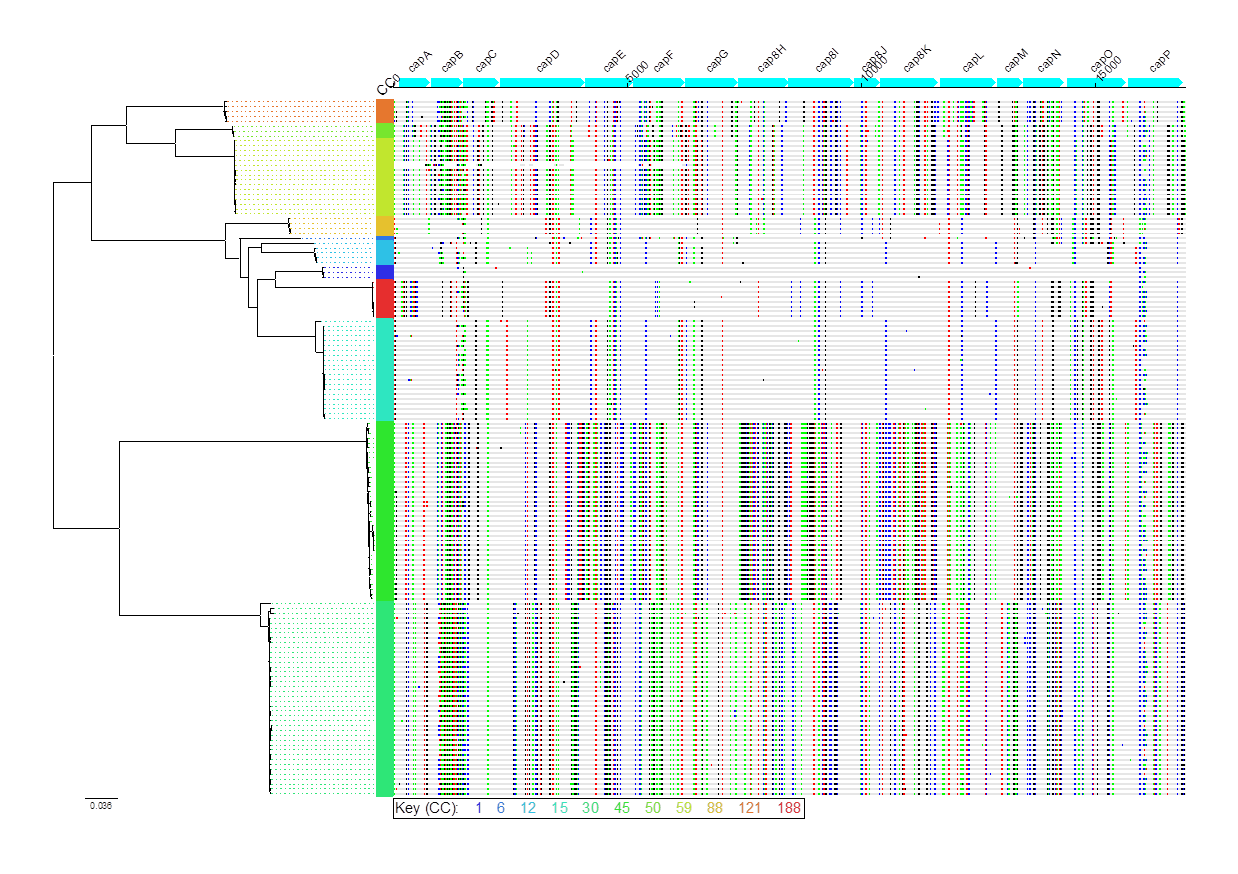


**B**

**C**


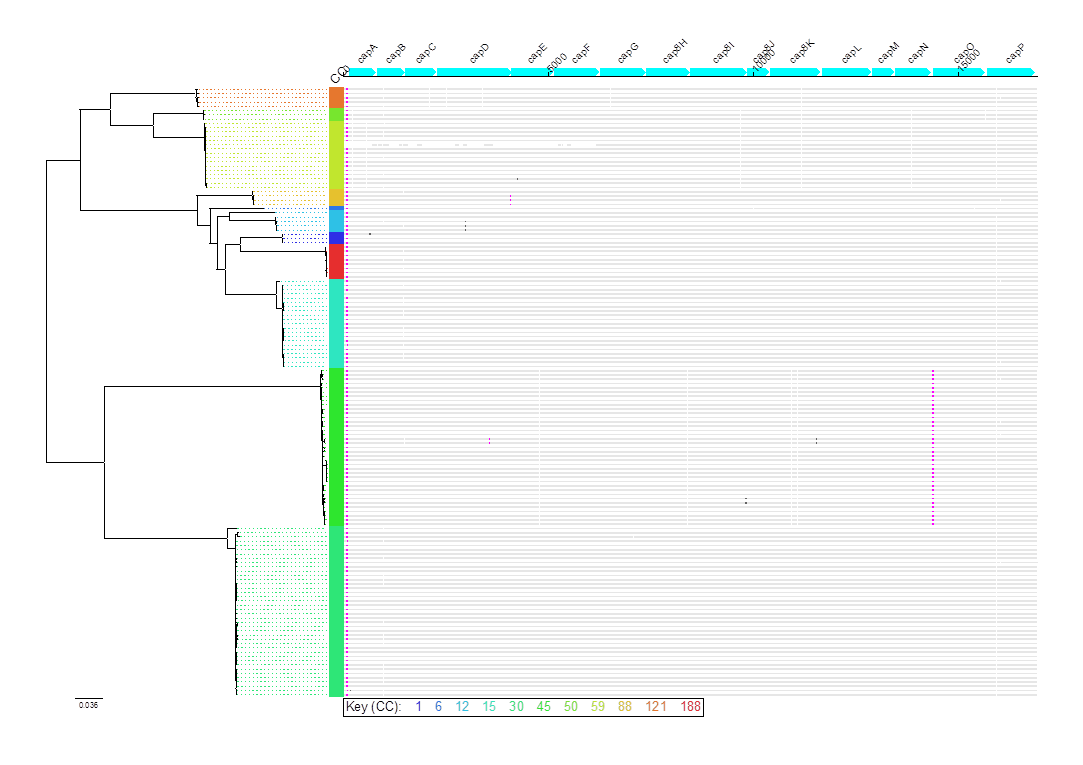


**D**

Supplement: S4 Fig — Approximately-maximum-likelihood phylogenetic trees of analyzed S. aureus CP5 (A and C) and CP8 (B and D) isolates annotated with the distribution of cap operon SNPs (A and B) and indels in US S. aureus isolates (C and D). (A) cap operon SNPs in CP5 isolates. SNPs were identified across the cap operon based on mapping against S. aureus HO 5096 0412 reference. Isolate tracks color-coded by CC (legend below figure). Color-coding of SNPs; Green A, Blue G, Black T and Red C. (B) cap operon SNPs in CP8 isolates. SNPs were identified across the cap operon and plotted against tree of all CP8 USA isolates based on mapping against S. aureus MSSA476 reference. Isolate tracks color-coded by CC legend below figure). (C) cap operon indels in CP5 isolates. Indels were identified across the cap operon and plotted against tree of all CP5 USA isolates based on mapping against S. aureus HO 5096 0412 reference. Isolate tracks color-coded by CC (legend below figure). Color-coding of indels; Vertical magenta dots indicates insertion while dark gray indicates deletion. For each isolate, the mapping coverage is indicated by the light grey horizontal field, with white regions demonstrating deletion of the corresponding cap genomic region. As such, this figure shows the partial loss of cap5D-E in a single CC97 isolate. (D) cap operon indels in CP8 isolates. Indels were identified across the cap operon and plotted against tree of all CP8 USA isolates based on mapping against S. aureus MSSA476 reference. (DOCX) [file pone.0208356.s007.docx]

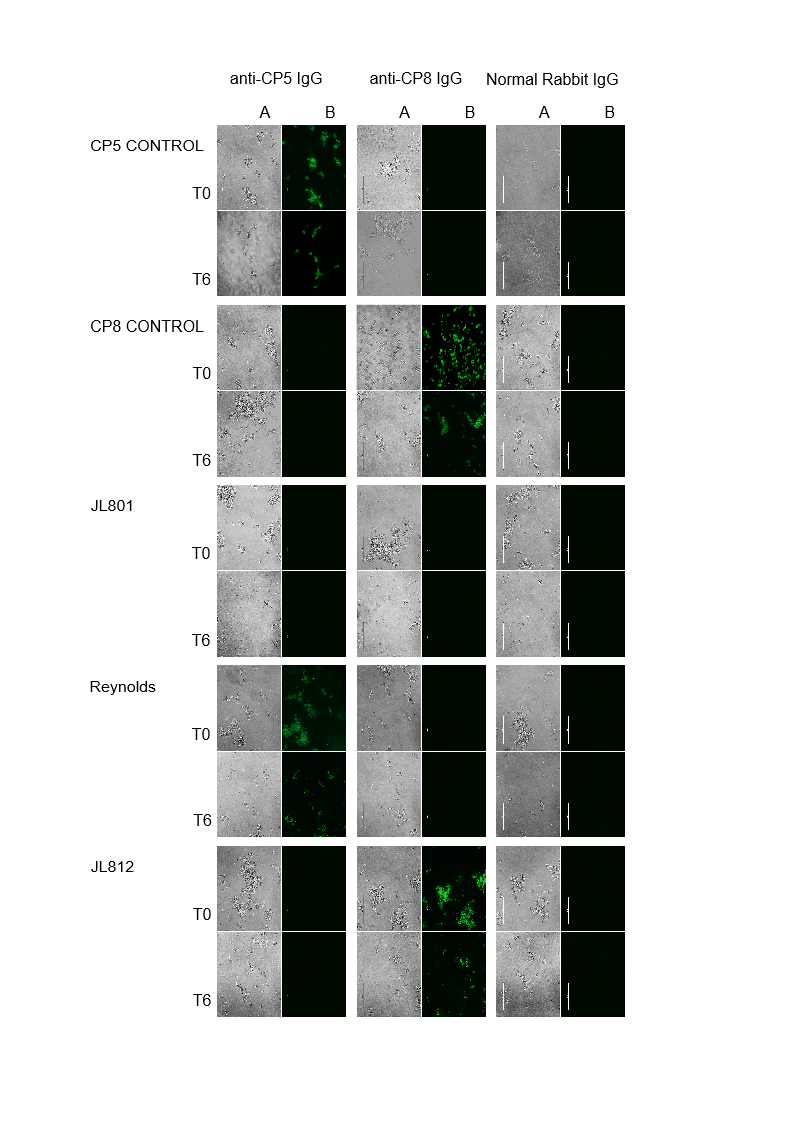

Supplement: S5 Fig — Three different S. aureus strains: Reynolds (CP5), its isogenic CP-negative mutant, and the CP- negative Reynolds complemented with CP8-coding sequences were used to challenge mice. The strains were tested in a blinded fashion with two independent experiments per strain. S. aureus was collected at the time of challenge (T0) and from the blood of infected mice 6 hr post infection (T6), and stained with rabbit anti-CP5, or rabbit anti-CP8 antibodies, or normal rabbit IgGs. Bright-field (A) and fluorescence photographs (B) of IFA staining are shown for each strain at both time points. (TIF) [file pone.0208356.s008.tif]

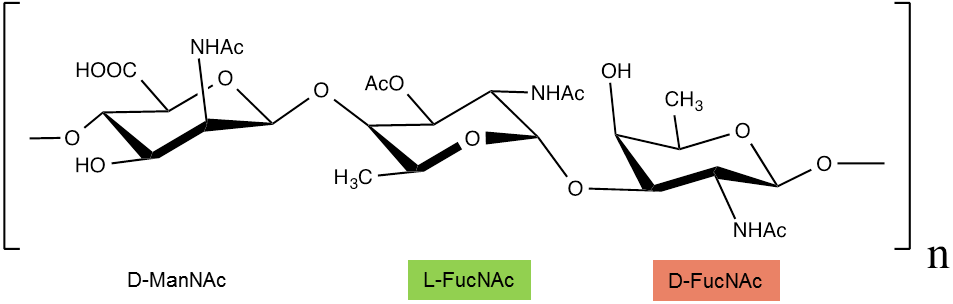


**A**

**B**

Supplement: S6 Fig — (A) Capsular polysaccharide type 5 is composed of repeat units of D-N-acetylmannosamine (D-ManNAc); L-N-acetylfucosamine (L-FucNAc) and D-L-acetylfucosamine (D-FucNAc). Cap5D is associated with synthesis of the D-FucNAc precursor (Li et al. Internatl. J. Med. Micro. 2014) and Cap5E is primarily associated with synthesizing L-FucNAc precursor (Miyafusa et al. FEBS Lett. 2013). (B) Cap5D is a 4, 6-dehydratase that converts UDP-D-GlcNAc to a D-FucNAc precursor, while Cap5E has 4, 6-dehydratase and 5-epimerase activity that converts UDP-D-GlcNAc to an L-FucNAc precursor but can also generate the analogous D-FucNAc precursor in a reverse epimerization reaction (Miyafusa et al. FEBS Lett. 2013). We propose that for S. aureus USA300 strains where the cap5D gene has a premature stop codon, the UDP-D-FucNAc precursor UDP-2-acetoamino-2, 6-dideoxy-a-D-xylo-4-hexulose (highlighted in orange) is derived as byproduct of the CapE epimerase reaction. (DOCX) [file pone.0208356.s009.docx]
