# Supplementary material for: Molecular epidemiology and expression of capsular polysaccharides in Staphylococcus aureus clinical isolates in the United States
Source: PLoS One. 2019 Jan 14;14(1):e0208356. doi: 10.1371/journal.pone.0208356 (PMC6331205; doi:10.1371/journal.pone.0208356)
Supplement: Supplementary file 5 [file pone.0208356.s006.docx]

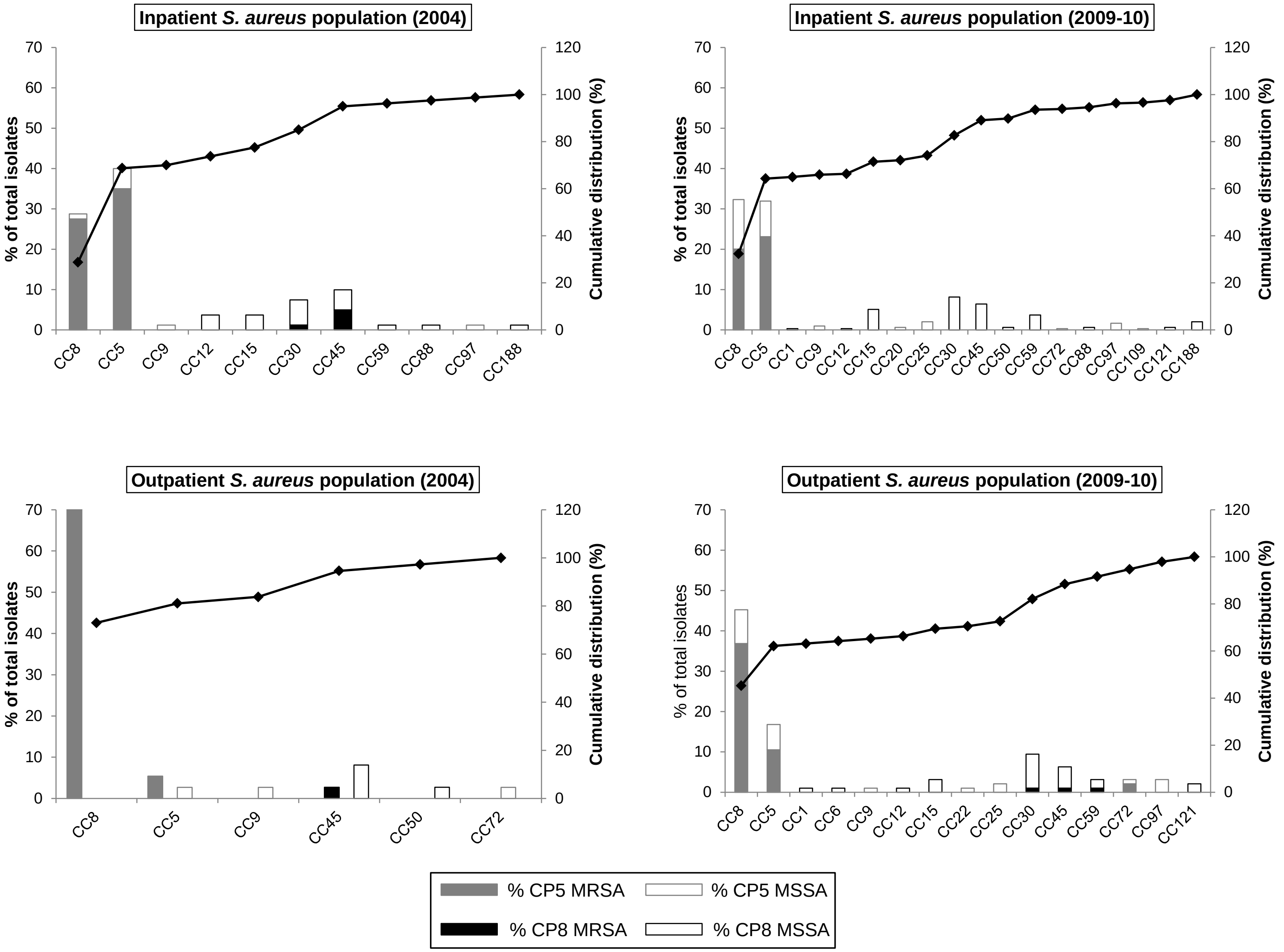
Inpatient S. aureus population (2004)
% of total isolates
Cumulative distribution (%)
CC8
CC5
CC9
CC12
CC15
CC30
CC45
CC59
CC88
CC97
CC188
Inpatient S. aureus population (2009-10)
% of total isolates
Cumulative distribution (%)
CC8
CC5
CC1
CC9
CC12
CC15
CC20
CC25
CC30
CC45
CC50
CC59
CC72
CC88
CC97
CC109
CC121
CC188
Outpatient S. aureus population (2004)
% of total isolates
Cumulative distribution (%)
CC8
CC5
CC9
CC45
CC50
CC72
Outpatient S. aureus population (2009-10)
% of total isolates
Cumulative distribution (%)
CC8
CC5
CC1
CC6
CC9
CC12
CC15
CC22
CC25
CC30
CC45
CC59
CC72
CC97
CC121
% CP5 MRSA
% CP5 MSSA
% CP8 MRSA
% CP8 MSSA
